# Nucleation of Stick‐Slip Instability Within a Large‐Scale Experimental Fault: Effects of Stress Heterogeneities Due to Loading and Gouge Layer Compaction

**DOI:** 10.1029/2019JB018429

**Published:** 2020-08-14

**Authors:** L. Buijze, Y. Guo, A. R. Niemeijer, S. Ma, C. J. Spiers

**Affiliations:** ^1^ High Pressure Temperature Laboratory, Department of Earth Sciences Utrecht University Utrecht The Netherlands; ^2^ Applied Geosciences, Energy Transition, TNO Utrecht The Netherlands; ^3^ State Key Laboratory of Earthquake Dynamics Institute of Geology, China Earthquake Administration Beijing China

**Keywords:** nucleation of stick‐slip instability, large‐scale experimental faults, gypsum gouge, rupture nucleation, PMMA

## Abstract

Geodetic observations and large‐scale laboratory experiments show that seismic instability is preceded by slow slip within a finite nucleation zone. In laboratory experiments rupture nucleation is studied mostly using bare (rock) interfaces, whereas upper crustal faults are typically filled with gouge. To investigate effects of gouge on rupture nucleation, we performed a biaxial shearing experiment on a 350 mm long saw‐cut fault filled with gypsum gouge, at room temperature and a minimum horizontal stress *σ*_2_ = 0.3–5 MPa. The gouge layer was sandwiched between polymethylmethacrylate (PMMA) plates For reference also a fault without gouge was deformed. Strain gauges and Digital Image Correlation were used to monitor the deformation field along the fault zone margins. Stick‐slip behavior occurred on both the gouge‐filled fault and the PMMA fault. Nucleation of instability on the PMMA fault persistently occurred from one location 2/3 to 3/4 along the fault adjacent to a slow slip zone at the fault end, but nucleation on the gouge‐filled fault was more variable, nucleating at the ends and/or at approximately 2/3 along the fault, with precursory slip occurring over a large fraction of the fault. Nucleation correlated to regions of high average fault stress ratio *τ/σ*_*n*_, which was more variable for the gouge‐filled fault due to small length scale variations in normal stress caused by heterogeneous gouge compaction. Rupture velocities and slip rates were lower for the gouge‐filled fault than for the bare PMMA fault. Stick‐slip persisted when *σ*_2_ was lowered and the nucleation zone length increased, expanding from the center to the sample ends before transitioning into instability.

## Key Points

- Nucleation of dynamic instability was observed along a 350 mm long experimental gouge‐filled fault sandwiched by compliant PMMA blocks
- Nucleation occurred in regions of high average stress ratio *τ/σ*_*n*_ but was more variable for a gouge‐filled fault than a bare PMMA fault
- The presence of gouge resulted in more heterogeneous fault stress, more precursory slip and lower rupture velocities

## 1 Introduction

Understanding the nucleation process of both natural and induced earthquakes is important, as it determines not only when, where, and how an earthquake starts, and also influences the ultimate size of a seismic event (Ellsworth & Beroza, 1995). The existence of a nucleation phase follows from the frictional weakening behavior that governs fault strength. Analytical and numerical studies have shown that nucleation on faults characterized by rate‐and‐state friction (RSF) (Dieterich, 1992; Rice, 1993; Rubin & Ampuero, 2005) or slip‐weakening friction (Campillo & Ionescu, 1997; Uenishi & Rice, 2003) requires a finite fault length that must slip aseismically before seismic instability can nucleate. This is termed the critical nucleation length, which is a function of the friction law, the frictional weakening parameters, the normal stress, and the elastic properties of the medium surrounding the fault (see further section 1.1). It is unclear how this nucleation length applies to earthquake nucleation in nature. For some natural earthquakes the nucleation process has been observed or inferred. For example, aseismic creep during the nucleation phase has been inferred from the occurrence of small repeating foreshocks near the hypocenters of intermediate and large earthquakes (Bouchon et al., 2011; Bouchon et al., 2013; Dodge et al., 1995; Dodge et al., 1996). Recently, aseismic slip accompanied by small foreshocks has been observed from combined geodetic and seismological measurements, prior to the occurrence of both a megathrust earthquake of M_w_ 8.1 (Ruiz et al., 2014; Socquet et al., 2017) and a M_w_ 6.9 event (Ruiz et al., 2017) at the Chile subduction zone in respectively 2014 and 2017. A slow nucleation phase was also recognized prior to a much smaller event of M_w_ 3.8 in Alaska (Tape et al., 2018). However, in many other cases the nucleation process could not be observed due to limited in situ geodetic data at sufficient spatiotemporal resolution. Improvement of understanding of the nucleation process could help to guide future instrumentation of earthquake‐prone faults to detect the rupture nucleation phase before earthquake rupture starts. This could potentially provide an early warning or forecast of the location and timing of natural events and of induced seismic events.

Laboratory experiments allow systematic investigation of the nucleation process and of the effects of loading conditions and fault properties on the size and duration of nucleation. In particular, in experiments using meter‐scale samples, dense instrumentation (e.g., strain gauges, piezo‐electric sensors, and displacement sensors) along a laboratory fault allows the faulting process to be studied in detail. Such experiments, typically performed at low normal stresses (<5 MPa), have shown how nucleation of an instability often starts as a localized slow slip zone covering a portion of the fault, which expands slowly at first and then accelerates rapidly leading to unstable rupture propagation (Latour et al., 2013; Ma et al., 2002; McLaskey & Kilgore, 2013; Ohnaka et al., 1986; Okubo & Dieterich, 1981; Zhuo et al., 2018). The observed nucleation zone in these tests was generally centimeters to meter‐scale (bare rocks) or millimeters to centimeters (polymers) in length scale. The expansion of the nucleation zone in an experiment using 1.5 m long granite blocks was accompanied by foreshocks indicating failure of highly stressed asperities within a creeping zone (McLaskey & Kilgore, 2013; Zhuo et al., 2018), similar to that observed in some natural fault zones. In accordance with theory (see section 1.1) the nucleation length was observed to increase with decreasing normal stress (e.g., Latour et al., 2013). When the nucleation length was close to the laboratory fault length, the mode of fault sliding (seismic vs. slow or aseismic slip) could be controlled by changing the loading conditions such as the normal stress (Mclaskey & Yamashita, 2017). Both experimental (Guérin‐Marthe et al., 2019; Kato et al., 1992; Xu et al., 2018) and numerical (Kaneko et al., 2016; Kaneko & Lapusta, 2008) studies further have shown that the nucleation length decreases with increasing loading rate, whereas it increases with fault roughness (Ohnaka & Shen, 1999; Yamashita et al., 2018).

Most of the above decimeter‐ to meter‐scale experiments investigating nucleation were performed using bare, smoothed surfaces of crystalline rocks, such as granite (e.g., Ohnaka & Kuwahara, 1990; Okubo & Dieterich, 1981) or granodiorite (Ma et al., 2002), or on polymers (e.g., Guérin‐Marthe et al., 2019; Latour et al., 2013). In nature, fault zones are usually filled with fault gouge or cataclastic rocks produced by wear of the formations flanking the fault. Many smaller scale experiments have focused on the friction of gouge, rather than bare rock surfaces (e.g., Ikari et al., 2011; Marone, 1998). Fault gouge may also be generated at asperities along initially bare rock surfaces but this requires a considerable amount of displacement—for example, several centimeters for smooth gabbro rocks (Yamashita et al., 2015). Gouge and bare rocks surfaces generally exhibit different frictional properties because the (time‐dependent) internal microstructure of the gouge, for example, can affect the strength, fault dilatancy, and the evolution of rate‐and‐state parameters with slip (e.g., Rathbun & Marone, 2013). These different frictional characteristics may lead to different nucleation behavior within gouge‐filled faults compared to bare rock faults.

Here, we study the nucleation process on gouge‐filled faults using simulated fault gouge sandwiched between two planar fault surfaces in a decimeter‐scale biaxial loading experiment. For the simulated gouge material, we used gypsum gouge; we conducted independent small‐scale triaxial experiments, which showed that gypsum gouge exhibits stick‐slip at room temperature and normal stresses <20 MPa. To be able to capture the nucleation process within the 350 mm long fault that was used in our experiments, we used polymethylmethacrylate (PMMA) forcing blocks which downscales the nucleation length. Polymer forcing blocks have a stiffness 20–25 times lower than rocks, such as granite, and have previously been used to simulate rupture nucleation in the laboratory. They have also been used in experiments that successfully reproduce earthquake phenomena such as the nucleation phase and supershear as well as crack‐like and pulse‐like rupture (e.g., Bayart et al., 2016; Lu et al., 2007). Rupture nucleation and propagation on the gouge‐filled fault was tracked using an array of strain gauges along the experimental fault, as well as (high‐speed) imaging of the PMMA blocks along the fault margin. The location of nucleation was considered in the light of the fault stresses that were observed from the strain data and obtained from Finite Element (FE) modeling.

### 1.1 RSF and Theoretical Nucleation Lengths

In this section RSF, conditions for instability, and several theoretical formulations for the nucleation length are presented, as well as relevant observations from numerical modeling and experiments. RSF is an empirical relationship widely used to describe fault frictional behavior, where friction *μ* is a function of slip velocity *V* and state *θ* (Dieterich, 1979; Ruina, 1983)

(1) $$\mu =\mu^{*}+\mathrm{aln}\frac{V}{V^{*}}+\mathrm{bln}\;\frac{V^{*}\theta }{D_{c}}$$

where *a* and *b* are constitutive rate‐and‐state parameters, *D*_*c*_ is the critical slip distance over which weakening (or strengthening occurs), μ* is a reference friction value, V* is a reference sliding velocity. Two equations for *θ* are predominantly used; the aging lawand the slip law

(2) $$\frac{\mathrm{d\theta}}{\mathrm{dt}}=1-\frac{\mathrm{V\theta}}{D_{c}}$$

and the slip law

(3) $$\frac{\mathrm{d\theta}}{\mathrm{dt}}=-\;\frac{\mathrm{V\theta}}{D_{c}}\;\ln\;\frac{\mathrm{V\theta}}{D_{c}}$$

At steady‐state sliding the parameter Ω *= Vθ/D*_*c*_ *=* 1 so *dθ/dt* = 0. For the aging law, *θ* increases at a sliding velocity of 0, whereas for the slip law θ can only change with slip.

Instability requires a negative *a* − *b* so that friction decreases with velocity (velocity weakening), whereas for a positive *a* − *b* friction increases with velocity which is stabilizing (velocity strengthening). For instability in, for example, a spring‐slider systems the spring stiffness *k* must be smaller than the critical stiffness *k*_*c*_ (= *σ*_*n*_(*b − a*)/*D*_*c*_). Note that for the slip law instability can also occur when *k > k*_*c*_ when the perturbation is large. Different formulations for the critical nucleation length exist, depending on the state law and weakening parameters, the sliding velocity, and the state of the fault (e.g., Ampuero & Rubin, 2008 ; Rubin & Ampuero, 2005). Equating the crack stiffness *k* (= 2G*/*πL*, where *L* is the 2‐D crack half‐length and G* is the 2‐D shear modulus which for out‐of‐plane strain is *G* and for in‐plane sliding *G*/(1 − *ν*), where *ν* is the Poisson's ratio) to the critical stiffness *k*_*c*_ gives a critical fault patch half‐length for instability to develop as *L*_*b‐a*_ = 2G*D_*c*_/*π*(*b* − *a*)*σ*_*n*_ (Rice, 1993; Ruina, 1983). For small perturbations this critical length is the same for the slip and the aging laws. Numerical modeling studies have shown that for velocities well over the steady state (Ω ≫ 1) a lower bound of the critical nucleation half‐length for the aging law is given by *L*_*b*_ = *G* * *D*_*c*_/(*bσ*_*n*_) (Dieterich, 1992). Linear stability analysis shows that for a slip zone at velocities close to steady state (Ω ~ 1) and *a*/*b* > 0.3781 an upper limit of the nucleation half‐length for the aging law is given by *L*_*∞*_ = (G * D_*c*_/*π*) (*b*/*(σ*_*n*_ (*b − a*)^2^)) (Ampuero & Rubin, 2008; Rubin & Ampuero, 2005). Recent simulations of nucleation on a velocity‐weakening patch show that nucleation in simulations with the aging law are better approximated by *L*_*∞*_. The modeled nucleation half‐lengths using the slip law are smaller and match better with *L*_*b‐a*_ (Chen & Lapusta, 2019). Linear stability analysis also shows the nucleation half‐lengths under the slip law are smaller than for the aging law, and also when a fault governed by the slip law is perturbed to values well above steady state the nucleation length may become several times smaller than expected from linear stability analysis (Ampuero & Rubin, 2008). Experimental data are often modeled with the slip or aging law to obtain the rate‐and‐state parameters. Several studies have shown that velocity steps (and, more recently, also slide‐hold‐slide steps) in experiments on bare rock or gouge are better described using the slip law (Ampuero & Ben‐Zion, 2008; Bhattacharya et al., 2015, 2017).

## 2 Experimental Materials and Methods

**Figure 1:**
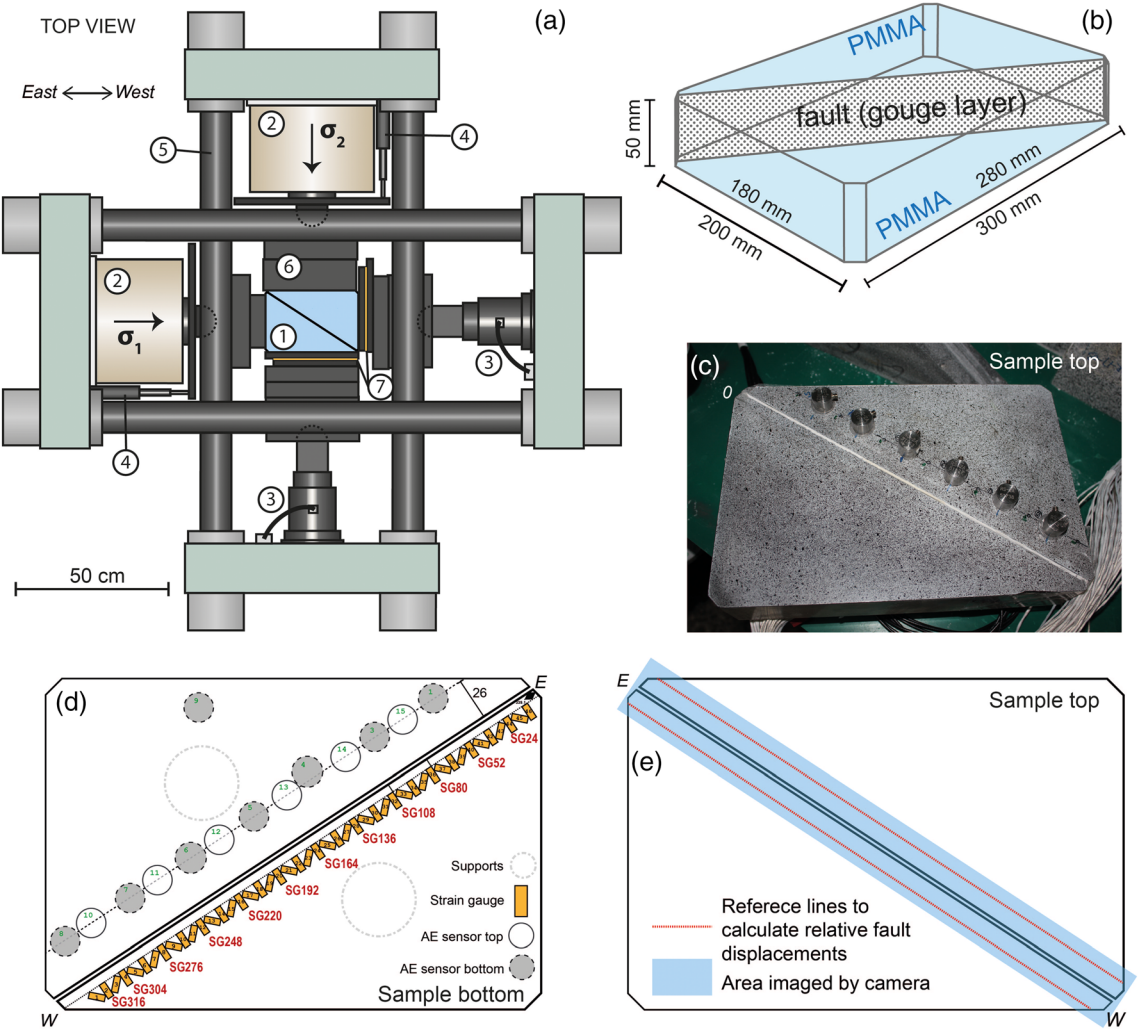
Biaxial deformation apparatus, sample assembly, and instrumentation. (a) Schematic drawing of the horizontal biaxial deformation rig, with 1: Sample assembly, 2: hydraulic presses, 3: load cell, 4: LVDT, 5: loading frame, 6: steel spacers, and 7: slide‐bearing steel plates. (b) Sample assembly. (c) Top of the sample assembly showing speckled surface and AE sensors. (d) Schematic drawing of the bottom of the sample assembly showing strain gauge configuration and AE sensors. E: east, W: west. (e) Schematic drawing of sample top showing the area imaged by the high‐speed camera, and the reference lines at 10 mm from the fault, which are used to compute relative fault displacements.

The experiments presented in this study were performed in the horizontal biaxial machine at the Institute of Geology, China Earthquake Administration in Beijing (Figure 1a). This horizontal rig allows biaxial loading of plate‐shaped samples up to 0.5 m in width, length, and/or thickness. The sample assembly used in this study (300 × 200 × 50 mm) was composed of two triangular forcing blocks, made of cast polymethylmethacrylate (PMMA), sandwiching a vertically oriented fault zone (347 × 50 mm) along the diagonal of the assembly. In two of the experiments the fault zone was filled with a gouge layer. For comparison, also an experiment without gouge was performed—that is, on a bare PMMA fault zone.

### 2.1 Fault Gouge Material and Frictional Properties

For the experiments using a gouge‐filled fault the fault zone was filled with a 2 mm thick gypsum gouge layer. This gouge was obtained by sieving crushed natural gypsum from the Paris Basin (de Meer & Spiers, 1997) to <150 μm. XRD analysis indicated that the crushed and sieved gypsum was 98.8% pure, with a small fraction of quartz (0.9%) and molybdenite (0.3%). Small‐scale, triaxial shearing experiments performed on a 2 mm thick layer of this gypsum gouge placed along the saw cut interface of 35 mm diameter PMMA forcing blocks, at a confining pressure of 5 MPa and load point velocities in the range 0.1–10 μm s^−1^ (Figure S1a in the supporting information), showed that the gypsum gouge had a friction coefficient of 0.75–0.85. Stick‐slip behavior was observed at all load point velocities (Figures S1b–S1d). Rate‐and‐state parameters for the gypsum gouge were obtained from velocity steps performed during separate friction experiments in a rotary shear apparatus and a biaxial machine, both using steel forcing blocks (Supporting Information S2). For the rotary shear apparatus (see, e.g., Van den Ende & Niemeijer, 2019) gypsum gouge was sheared under normal stresses between 2 and 10 MPa and load point velocities of 5–100 μm s^−1^. The velocity steps in the rotary shear apparatus could be modeled with a single state variable, which yielded *a − b* in the range −0.0004 to −0.006 with an average value of −0.0031 and a *D*_*c*_ of 1–7 μm with an average of 2.9 μm (Figure S3). In the biaxial experiments gouge was sheared at a normal stress of 10–13 MPa, loading rates 3–1,000 μm s^−1^ and 100% relative humidity (RH) (C. Marone, personal communication August 22, 2018). The velocity steps in the biaxial apparatus were best modeled with a two‐state variable friction law, which can model an initial rapid drop in friction, as well as a longer term weakening trend (e.g., Tullis & Weeks, 1986). The fits yielded the following rate‐and‐state parameters for the aging law: *a − b* = −0.003 to −0.005, *b*_1_ = 0.002–0.005, *b*_2_ = 0.001–0.0035, *D*_*c*1_ = 3–7 μm, and *D*_*c*2_ = 50–150 μm, and a steady state friction coefficient of 0.8.

### 2.2 Sample Assembly, Deformation Apparatus, and Experimental Procedure

The PMMA forcing blocks (#1 in Figure 1a) were manufactured by WSV Kunststoffen BV. The elastic properties provided or these blocks were a (static) Young's modulus *E* of 3.21 GPa, Poisson's ratio *ν* of 0.37, and a density *ρ* of 1,190 kg m^−3^. The shear wave, and *P* wave velocities measured on PMMA blocks by Svetlizky and Fineberg (2014) and Bayart et al. (2016) were respectively *V*_*s*_ = 1,345 m s^−1^, and *V*_*P*_ = 2,700 m s^−1^, which give a Rayleigh wave velocity *V*_*R*_ = 1,237 m s^−1^ under plane stress conditions. The corresponding high‐strain‐rate or dynamic Young's modulus computed from these velocities was 5.6 GPa, which is significantly larger than the static value of 3.2 GPa mentioned before. The fault surfaces of the forcing blocks were prepared by manual grinding #80 SiC powder. To prepare the gouge layer, the fault surface of one of the PMMA blocks was rotated to a horizontal position. The fault gouge (see section 2.1) was wetted and distributed evenly along the fault surface with a thickness of 2 mm and was left to dry at ambient conditions. Afterwards the block and gouge layer were equilibrated to a RH of 75% in an enclosed container for >1 day. The RH in the container was maintained by a saturated NaCl solution. Subsequently, the block and gouge layer were taken out of the container, and the second PMMA block was then added so that the gouge layer was sandwiched by the two PMMA blocks, thereby minimizing exposure of the gouge to ambient conditions. Tape was applied at the lower fault margin and fault ends to hold the sample assembly together and prevent gouge from extruding. The sample assembly was rotated and placed horizontally in the biaxial machine (#1 in Figure 1a). The assembly was supported at the base by two steel cylinders of 50 mm in diameter. Slide‐bearing plates (#7 in Figure 1a) were placed along the sides of the sample assembly to accommodate lateral motion as the blocks were displaced along the fault zone. Steel spacers blocks (#6 in Figure 1a) bridged the gap between the hydraulic presses and the slide‐bearing plates.

Testing of the experimental fault zones containing gouge commenced with a load‐controlled precompaction phase, subjecting the sample assembly to horizontal stresses *σ*_1_ and *σ*_2_ of 20 MPa for 10 min. The principal stresses *σ*_1_ and *σ*_2_ were then simultaneously reduced to 5 MPa. For the bare PMMA fault the precompaction phase was skipped and the sample was directly loaded to 5 MPa. Subsequently, shear displacement was imposed along the fault by switching loading in the *σ*_1_ direction to displacement control, while keeping loading in the *σ*_2_ direction under load control. This shear phase started with a run‐in stage where the hydraulic press in the *σ*_1_ direction was advanced at fixed load point velocity *v*_1_ of 5 μm s^−1^, increasing *σ*_1_ while retaining the minimum horizontal stress or “confining stress” *σ*_2_ = 5 MPa. After a load point displacement *d*_1_ of 3 mm the load point velocity was reduced to 1 μm s^−1^ for a load point displacement of 1.5 mm. Subsequently, for one of the gouge‐filled faults (hbr‐17‐19) and the bare PMMA fault *σ*_2_ was changed stepwise to vary the critical stiffness and the critical nucleation length (see section 1.1), which could affect the sliding behavior. To step *σ*_2_, first, the load point displacement in the *σ*_1_ direction was halted, *σ*_2_ was changed to the desired value, and then loading in the *σ*_1_ direction was reinitiated. The range of *σ*_2_ evaluated was 0.3–5 MPa, starting with reduction of *σ*_2_ and then increasing *σ*_2_ toward the end of the experiment. For upsteps in *σ*_2_ the load point velocity was first set to 5 μm s^−1^ and reduced to 1 μm s^−1^ when steady state was reached. For each value of *σ*_2_ a total load point displacement of 0.5–1 mm was imposed at a load point velocity of 1 μm s^−1^. The cumulative load point displacement over the experiment was 10 mm. The control experiment without gouge followed the normal confining stress stepping procedure. A second experiment with gouge (hbr‐17‐18) was performed, where only sliding at *σ*_2_ = 5 MPa was performed, up to 4.5 mm of load point displacement.

### 2.3 Data Acquisition and Processing

The mechanical data acquired include the orthogonal loads applied by the hydraulic presses (#2 in Figure 1a) and the load point displacements. Two load cells (#3 in Figure 1a) measured the loads applied to the sample to a resolution of ±3 kg, yielding the principal stresses *σ*_1_ and *σ*_2_ (±0.003 MPa) applied at the outer sample edges. Using the sample geometry, these stresses were translated into the normal stress *σ*_*n*_ * and shear stress τ* acting on the fault surface, where * is used to denote macroscopic, or far‐field, values on the fault. The ratio between these stresses gives the macroscopic fault friction μ* = τ*/σ_*n*_ *. The load point displacements *d*_1_ and *d*_2_ were measured using two linear variable displacement transducers (LVDTs) situated between the frame and a metal bar attached to the hydraulic presses (Figure 1a #4) to within ±0.25 μm. The macroscopic imposed displacement along the fault can be expressed as *d*_*t*_ * = d_1_ */cos θ* ≈ 1.2 *d*_1_. Both the loads and the load point displacements were recorded continuously at 1 kHz. The mechanical data were filtered with a Savitsky‐Golay filter to remove high frequency noise yet retain the rapid changes during stick‐slip events.

Strains along the fault were measured using 46 single‐component metal foil resistance strain gauges BE120‐5AA (HT Sensor Technology Co. Ltd.), which have dimensions of 9 × 4.5 mm (grid length 5 mm). The strain gauges were glued on the base of one the PMMA forcing blocks along the fault zone, with the center of each gauge located 10 mm from the fault zone (Figure 1d). The gauges were oriented at angles alternating between 45°, 90°, −45°, and 90° to the fault zone, thus forming 22 rectangular strain gauge rosettes. The rosettes are labeled SG24 through SG316, with the number indicating the along‐fault distance of the rosette center from the eastern end of the fault in mm. The maximum frequency *φ*_*c*_ that can be recorded the strain gauges is determined by their length *L*_*g*_ (5 mm) with respect to the wavelength of the longitudinal waves, through *φ*_*c*_ *= V*_*P*_ */(n L_*g*_)*. For the current setup frequencies up to 250 kHz would be detected by the gauges (*n =* 2), but for good resolution of the recorded waveforms a higher value for *n* is required, for example, *n =* 10, which would yield a *φ*_*c*_ of 50 kHz. The strain gauge signals were conditioned by a 28,000 Signal Conditioning system with 28454A Quad‐channel conditioners (Precision Filters Inc.), with a cut‐off frequency of 102.3 kHz. Strain signals were recorded continuously at 500 kHz at a 16‐bit resolution by a DS‐128 High Speed Data Acquisition Instrument (Beijing Softland Times Scientific & Technology Co. Ltd.). Four gauges malfunctioned during the experiment, causing gaps in the strain data at some locations. Strain gauge recordings were filtered with a Savitsky‐Golay filter. From the strain gauge rosettes, the 2‐D strain tensor can be derived (see Supporting Information S3), which can be used to compute the local shear stress *τ* and local normal stress *σ*_*n*_ with respect to the fault orientation (Equations S10 and S11). From the changes in shear stress the nucleation process can be observed. We consider the nucleation zone as the fault length that was slipping slowly before the slip zone expanded at a steady rupture velocity of >5% of the Rayleigh wave speed. We consider the local stress drop Δ*τ* as the difference between the shear stress 0.1 s before the slip event and the mean shear stress 1–2 ms after the slip event.

The top surfaces of both PMMA forcing blocks were painted white with a black speckle pattern (Figure 1c), which was used for Digital Image Correlation (DIC). During the experiments the top surface was imaged by a Photron Fastcam SA2 high‐speed camera, which was suspended in a frame above the sample. LED lights were used to illuminate the sample surface. Images (2,048 × 228 pixels) of a 350 × 39 mm area centered around the fault zone (1 pixel = 0.171 mm for the gouge‐filled faults, 1 pixel = 0.24 mm for the PMMA fault) were acquired at 1 Hz during parts of the experiment (Figure 1e). Selected slip events were imaged at frame rates up to 9,000 fps. Relevant image data were processed using the freely available PIVLab v 2.02 (Thielicke & Stamhuis, 2014) to obtain the displacement field around the fault. The smallest window size that was used for the image processing was 22 pixels (gouge‐filled fault) or 16 pixels (PMMA fault), and the resolution in displacement was ±5 μm (Zhuo et al., 2018). The displacement field was expressed in a fault‐normal or a fault‐parallel direction, with respect to a given starting situation (e.g., start of shearing). To compute the relative fault‐normal displacement *d*_*n*_ (positive is compaction) and fault‐parallel displacement *d*_*t*_ (positive is left‐lateral motion), the displacements along two lines parallel to the fault zone at a distance of 10 mm from the fault were subtracted (Zhuo et al., 2018). The maximum slip rate was determined as the maximum difference in shear displacement between image frames. The fault‐normal displacement could be interpreted as compaction of the fault zone, assuming the fault‐normal elastic deformation of the PMMA + the fault zone was small relative to the deformation of the fault zone itself. The shear displacement *d*_*t*_ recorded between slip events at 1 fps was corrected for the elastic deformation in the PMMA forcing blocks. The was done by fitting the shear stress rate from the strain data between the slip events, and interpolating this rate along the fault zone. For each DIC frame the cumulative elastic shear strain was then computed by multiplying the shear stress rate with the cumulative time and dividing by the shear modulus of PMMA. To obtain the corrected relative shear displacement, the shear strain was then multiplied with 20 mm and subtracted from the total relative shear displacement.

## 3 Results

Here we summarize the experimental results, including the far‐field mechanical data, the local stresses recorded by the strain gauge array, and the deformation field obtained from Digital Image Correlation. We focus on the experiment with the gypsum fault (hbr‐17‐19) but show several results for the second experiment with a gypsum fault and the experiment with a bare PMMA fault for reference.

### 3.1 Macroscopic Fault Stresses and Apparent Friction

**Figure 2:**
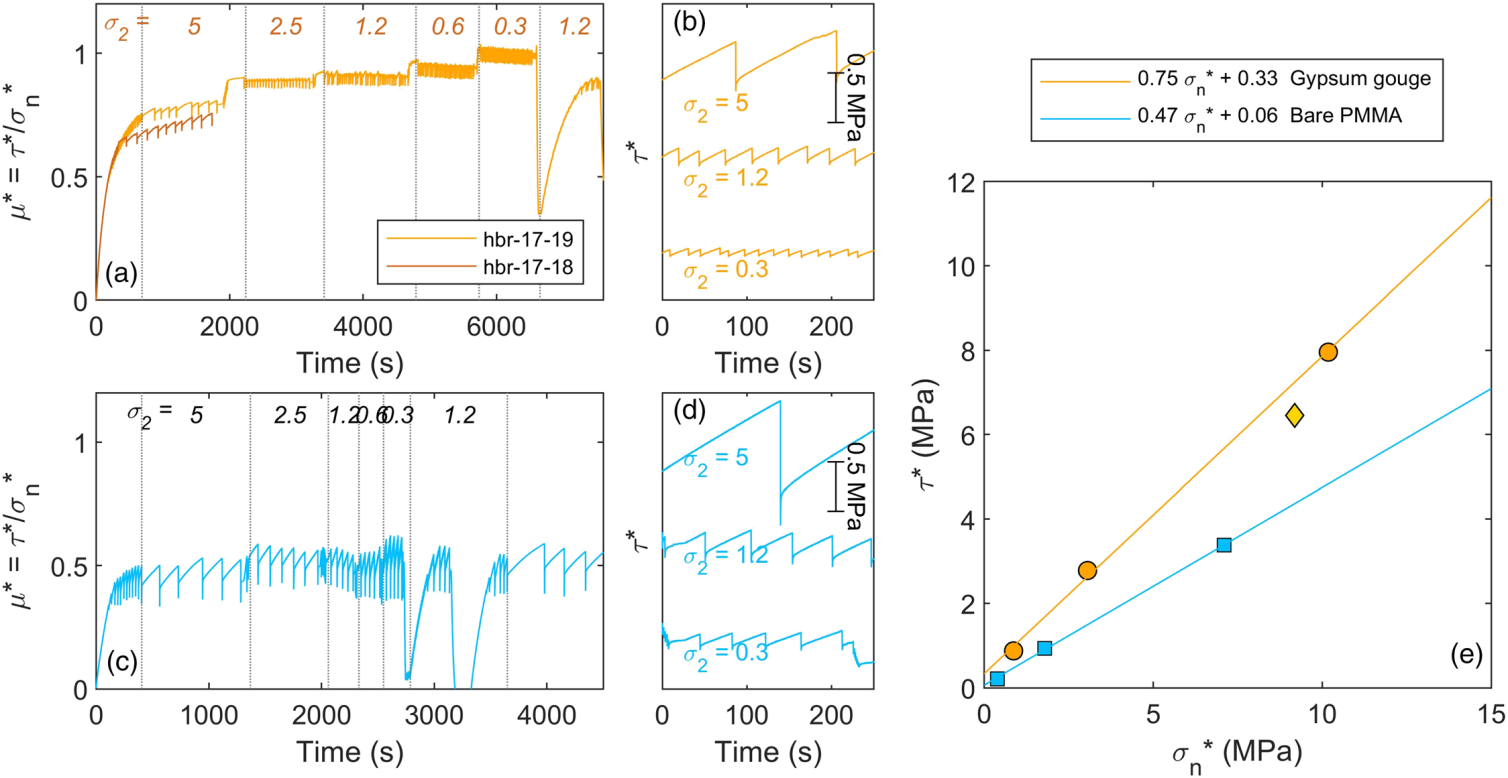
Macroscopic stresses measured during sliding on the gypsum faults (hbr‐17‐18 and hbr‐17‐19) and a bare PMMA fault (hbr‐19‐31). Macroscopic friction coefficient μ* (i.e., the ratio of the macroscopic shear and normal stresses τ*/*σ*_n_*) against time since start of shearing for (a) gypsum gouge faults, and (c) bare PMMA fault. The italic numbers indicate the value of constant stress boundary *σ*_2_ in MPa. The first dotted line at ~500s indicates when the sliding velocity was stepped down from 5 to 1 μm s^−1^. Zoom in of macroscopic shear stress τ* during stick‐slip events at a confining stress *σ*_2_ = 5, *σ*_2_ = 1.2, and *σ*_2_ = 0.3 MPa for (b) gypsum gouge faults and (d) bare PMMA fault. The magnitude of the macroscopic shear stress τ* is indicated by the scale bar. (e) Macroscopic shear and normal stresses for gypsum gouge fault and bare PMMA fault.

The evolution of macroscopic friction τ*/*σ*_n_* with time showed rapid initial hardening, followed by “yielding” toward a value of 0.75–0.8 for both gouge‐filled faults (Figures 2a and 2e) or 0.45 for the bare PMMA fault (Figure 2c). After a *d*_1_ of 4.5 mm the confining stress *σ*_2_ was decreased stepwise for one of the gouge‐filled experiments (hbr‐17‐19), during which the macroscopic shear stress decreased stepwise to reach a minimum value of 0.6 MPa at *σ*_2_ = 0.3 MPa (Figures 2a and 2b). Fitting the average macroscopic shear stress τ* and normal stress *σ*_*n*_ * for the different stress steps with the Mohr Coulomb criterion gives a friction coefficient of 0.75, and a cohesion of 0.33 MPa (Figure 2e). The confining stress was also varied on the bare PMMA fault, and the measured macroscopic stresses indicated a lower friction coefficient of 0.47.

For both the gouge‐filled faults and the bare PMMA fault stick‐slip events persisted throughout the experiment at all *σ*_2_ steps. For the gouge‐filled fault the associated macroscopic stress drop Δτ* was 0.4 MPa at the highest stress (*σ*_2_ = 5 MPa), with a recurrence interval of ~120 s (Figure 2b). During some stick‐slips cycles, smaller friction drops were observed prior to the main stick‐slip event. The macroscopic stress drop decreased from 0.4 to 0.1 MPa at the lowest stresses, that is, at *σ*_2_ = 0.3 MPa. The corresponding recurrence interval decreased from 120 to 16 s. The stick‐slips were audible at *σ*_2_ of 5, 2.5, and 1.2 MPa but became inaudible at lower stresses. For the bare PMMA fault the Δτ* was larger, exceeding 1 MPa with a recurrence interval of 200 s (Figure 2d). At lower *σ*_2_ the stress drop decreased to 0.3 and 0.1 MPa.

### 3.2 Local Fault Stresses and Slip at *σ*_2_ = 5 MPa

**Figure 3:**
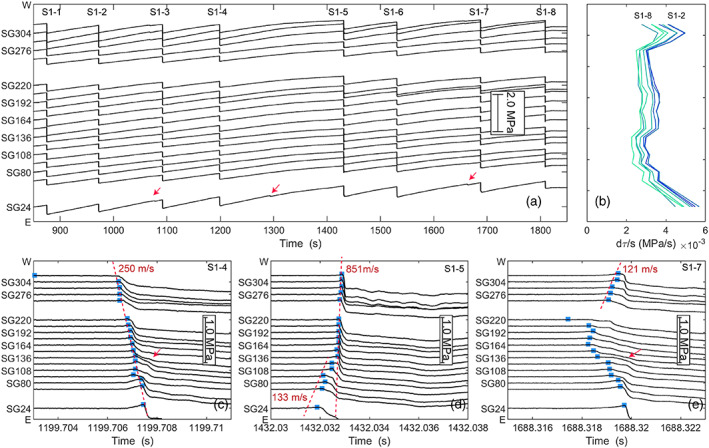
Overview of local shear stresses obtained from the strain gauge measurements during stick‐slip events observed the gypsum fault (hbr‐17‐19) during sliding at a confining stress *σ*_2_ of 5 MPa and a load point velocity *v*_1_ of 1 μm s^−1^. The locations of the strain gauges are indicated by the SG labels along the y‐axis, where the number indicates the position in mm with respect to the eastern end (E). The magnitude of the stress change is given by the scale bar. (a) Local shear stresses 850–1850 s (load point displacement 3.7–4.2 mm). Red arrows indicate small events only rupturing the fault ends, (b) shear stress rate between stick‐slip events. This rate is obtained by linear fits of the shear stress over the time interval 2 s after the previous events to 2 s before the next event. (c–e) Zoomed‐in view of local shear stresses during stick‐slip events S1‐4, S1‐5, and S1‐7. Blue markers indicate peak shear stresses. The dotted lines show fits of the peak stresses, which give rupture velocity *v*_*r*_. Red arrows indicate the secondary rupture front propagating back across the fault once rupture or nucleation reaches the fault end.

Local shear stresses *τ* along the fault margin were obtained from the strains measured by the strain gauge array plus elastic properties of the PMMA blocks (section 2.3). Figure 3a shows the local shear stress changes during the first loading stage following the run‐in (*σ*_2_ = 5 MPa, *v*_1_ = 1 μm s^−1^). The local shear stress changes clearly show the stick‐slip events, which were also visible in the macroscopic mechanical data (Figure 2). Slip events occurred at regular intervals of ~120 s, apart for Event S1‐5, which had a recurrence interval of 230 s. The largest stress drops were observed near the ends of the fault. Several smaller stress drop events occurred near the ends of the fault between the main stick‐slip events, for example, at 1080s, 1190s, and 1670s. The increase in local shear stress due to loading was not uniform along the fault zone; near the eastern end (i.e., at the side where loading is applied) the shear stress increase was larger at the fault ends than in the fault center and was largest near the eastern end of the fault (Figure 3b). The local shear stresses between stick‐slip events increased in a near‐linear manner in response to loading imposed by the machine, but in particular, near the fault ends the shear stress increase deviated from the linear trend. Such deviations indicate the onset of precursory slip related to nucleation of stick‐slip instability (see, e.g., Dieterich, 1992; McLaskey & Kilgore, 2013; Ohnaka et al., 1986; Yamashita et al., 2018), as slip relaxes the shear stresses in the medium around the fault.

A closer inspection of the shear stresses recorded during the stick‐slip events shows that nucleation of (fast) slip starts from either end of the fault zone (Figures 3c and 3d) or from a broader zone near the center of the fault (Figure 3e). For the events nucleating at the ends the local shear stress was observed to decrease prior to the stick‐slip at the gauge locations closest to the end indicating slow slip, for example, at SG318 in case of Event S1‐4 (Figure 3c). This slow slip zone then accelerated into a dynamic event propagating from SG304 to the other end of the sample at an average rupture velocity of 250 m s^−1^, that is, 0.2 times the Rayleigh wave speed *V*_*R*_ (= 1,237 m s^−1^). The size of the nucleation zone for this event was thus smaller than ~40 mm (distance from western edge to SG304). For Event S1‐5, yielding started at SG24 and the slip zone first expanded at a slow rupture velocity *v*_*r*_ of 133 m s^−1^ (0.11 *V*_*R*_) up to SG124 before transitioning into a faster rupture propagating at 851 m s^−1^, or 0.68 *V*_*R*_ (Figure 3d). For all events, local accelerations and deceleration of rupture velocity were observed. The rupture velocity was typically fastest in the center of the fault (SG136–SG220) and the west end of the fault (SG262–SG 318), whereas rupture usually decelerated between SG108–SG136 and SG220–SG262, as can, for example, be seen for Event S1‐4 (Figure 3c).

Other slip events nucleated from a position closer to the central portion of the fault at about two thirds from the eastern end (SG192–SG220). Some of the events were preceded by a smaller slip event, which nucleated at the fault end but was arrested. For Event S1‐7 for example a small event was observed 0.07 s prior to the main event, which started at the western end but was arrested between SG262 and SG220 (not shown). This event caused a gradual decrease in local shear stress (local yielding) between SG136 and SG192 indicating that slow slip accelerated in the central portion of the fault (Figure 3e). At 1688.318 s the slow slip zone accelerated from between these two slip zones from SG220 toward the ends of the fault reaching a rupture velocity of ~120 m s^−1^, 0.1 *V*_*R*_. Upon reaching one of the fault ends a secondary rupture front propagated back along the fault resulting in a large stress drop at the ends and further stress release in the center (Figure 3e). This rupture velocity of the secondary front was faster than for the primary rupture, but it was not resolved well enough to obtain the rupture velocity.

**Figure 4:**
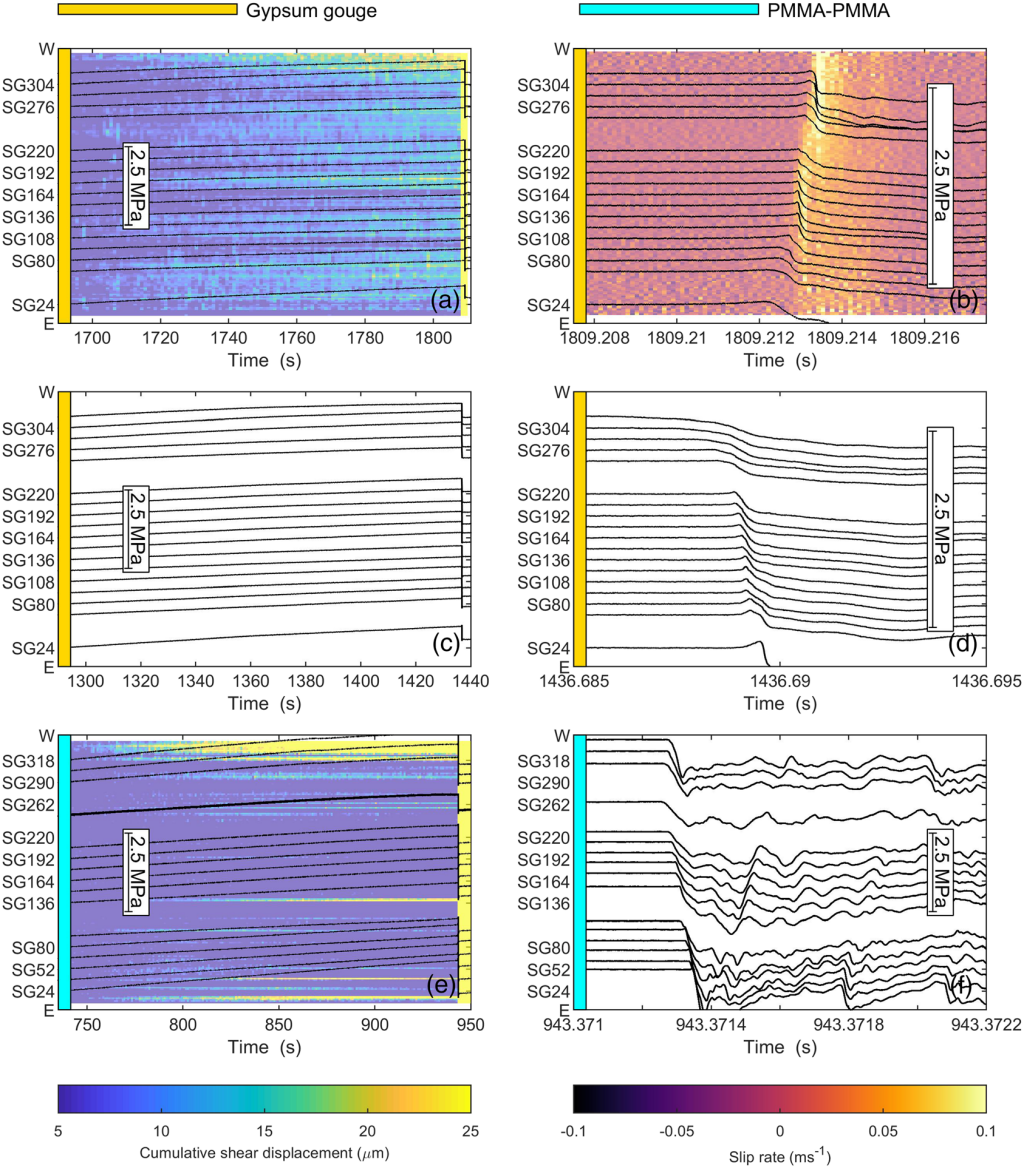
Comparison of local shear stress changes and slip during slip events on a gypsum fault (experiments hbr‐17‐19 and hbr‐17‐18) and on a bare PMMA fault (experiment hbr‐19‐31). All results are obtained at *σ*_2_ = 5 MPa and a load point velocity of 1 μm s^−1^. Background colors give cumulative shear slip or the slip rate obtained from Digital Image Correlation, where available. Note that the cumulative shear slip is truncated at 30 μm to highlight slip occurring prior to the slip events. (a) Interseismic period following Event S1‐7 up to Event S1‐8 (Figure 3a), (b) zoomed‐in view of shear stresses and slip rates for Event S1‐8, (c) shear stresses during the interseismic period for the second gypsum fault experiment (hbr‐17‐18), (d) zoomed‐in view of the shear stress changes of the event shown in (c), (e) shear stresses and cumulative slip during the interseismic period for the bare PMMA fault, and (f) zoomed‐in view of shear stresses during the slip event shown in (e).

Digital Image Correlation was used to analyze the fault shear and normal displacements for the last event in Figure 3a (S1‐8), which nucleated at the east end of the fault. Image data acquired at 1 fps show precursory slip occurred over almost the entire interseismic period on a large fraction of the fault (Figure 4a). Consistent with the nonlinear stress increase visible in the stress traces near the western fault end in Figure 3a, DIC showed most precursory slip occurred near the western fault end, amounting to 20–25 μm just prior to the slip event. However, also in the center of the fault (SG164–SG192) 15–20 μm of slip was observed, whereas less slip was observed on the eastern half of the fault, for example, between SG80 and SG164. At 1809.213 yielding of the shear stress showed that an ~100 mm wide slip zone near the east end accelerated and transitioned into a fast slip event that propagated toward the west end (Figure 4b). Rupture decelerated around SG220–SG276 but accelerated again as the west end of the fault ruptured. This is consistent with the decelerations seen in the strain gauge data in, for example, Figures 3c and 3d. The slip obtained from DIC indicated a total shear slip of 100 μm, and the slip rate ranged up to 0.1 m s^−1^, which is a lower bound estimate as the sampling interval of the images was 0.11 ms. The average rupture velocity of this event was 491 m s^−1^, but locally rupture propagated faster, attaining supershear velocities approaching *V*_*P*_ in the center of the fault.

For comparison a second experiment was performed on a gypsum fault, and a third experiment on a fault without gouge (bare PMMA surfaces). For the second gypsum experiment less variability was observed for the nucleation site than for the experiment shown in Figure 3. Most events nucleated from the western end of the fault (e.g., Figures 4c and 4d), but some events also nucleated from approximately two thirds of the fault near SG220 (not shown). Rupture velocities were somewhat faster with 190–400 m s^−1^ for the events nucleating from the ends, and 150 m s^−1^ for the events nucleating from SG220. Contrary to the other gouge experiment hbr‐17‐19, no events nucleated near the eastern end. Note however that for both experiments the total number of stick‐slip events at *σ*_2_ = 5 MPa was limited; it is possible that less variability in nucleation location would be observed for larger load point displacements.

**Figure 5:**
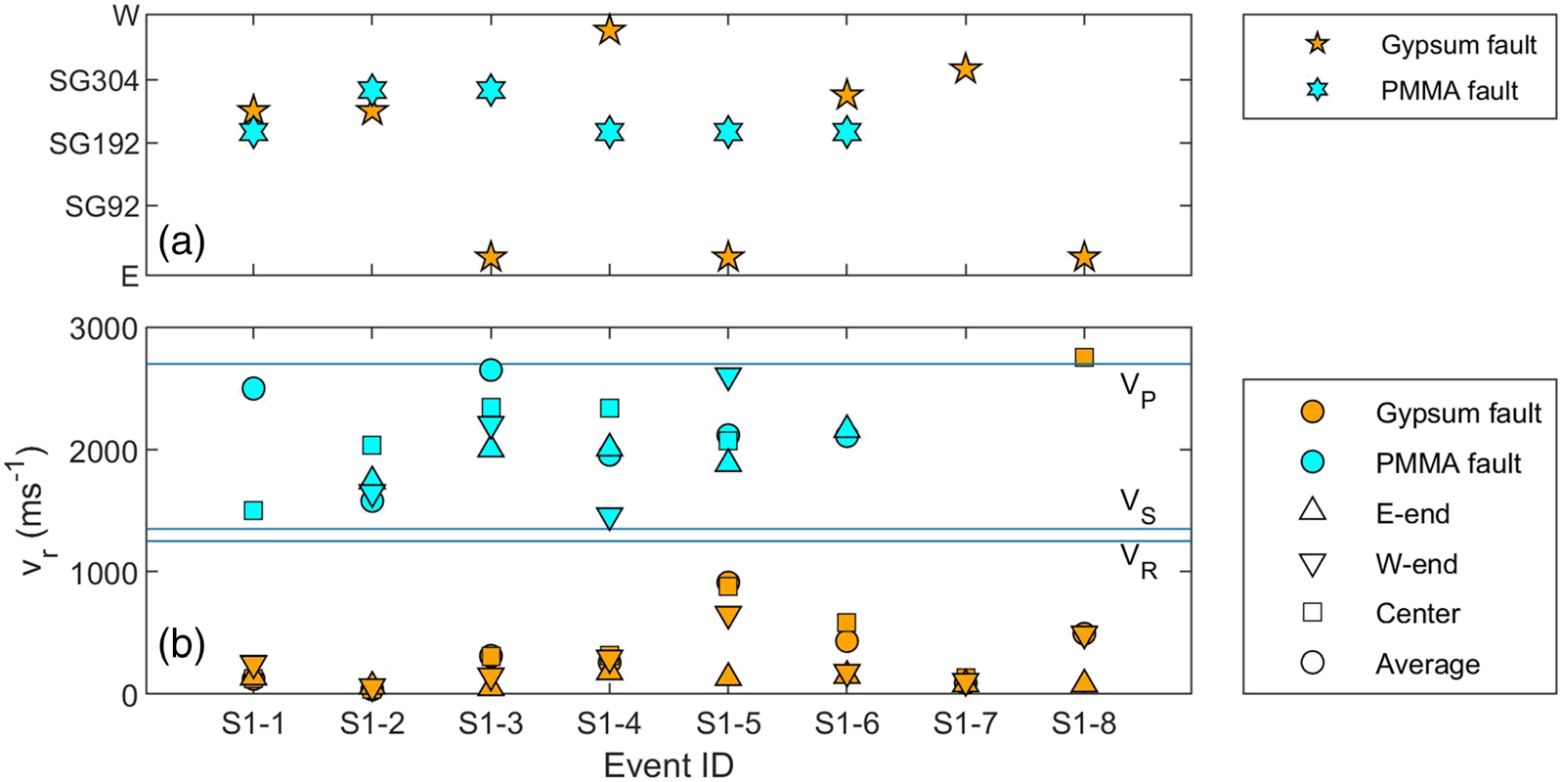
Nucleation site and rupture velocities *v*_*r*_ measured on the gypsum gouge faults and the bare PMMA fault. (a) Nucleation site for the different faults given as distance along the fault (sensor locations as in Figure 3). The nucleation site is taken as the strain gauge location at which slow yielding was first observed, or, in case no such slow slip zone is recognized, the point from which rupture accelerates. (b) Rupture velocities measured by fitting peak shear stresses during rupture. The average rupture velocity (squares) is the rupture velocity measured after rupture propagates at a constant velocity, or, for events nucleating from the center, the average velocity with which the slip zone propagates toward the fault ends. The rupture velocity is also determined for the east end, west end, and center, where the rupture velocity is averaged between respectively SG24–SG122, SG248–SG318, and SG136–SG220. *V*_*P*_, *V*_*S*_, and *V*_*R*_ indicate respectively the *P* wave speed, shear wave speed, and Rayleigh wave speed of PMMA.

#### 3.2.1 Comparison Between Gouge‐Filled Faults and Bare PMMA Fault

Slip events on the PMMA fault showed different behavior compared to those observed on the gypsum gouge fault. Even though the recurrence time and stress build‐up were almost twice as large as on the gouge‐filled fault, less precursory slip was observed on the PMMA fault (almost none in the middle of the fault) except for the western end where up to 45 μm of slip was observed in the interseismic period (Figure 4e). Events always nucleated from the same location, accelerating from ~SG192–SG248, which is adjacent the precursory slip zone at the W‐end of the fault (Figure 4f). This location is similar to the location from which the center events nucleated on the gypsum fault (Figure 5a).

Image data (2000 fps) indicated a total shear slip of 160–200 μm was achieved along the fault over the slip event. Assuming most of this slip occurred within the duration of the main slip event shown in Figure 4f (~0.05 ms), slip rates may have exceeded several meters per second. Rupture propagation on the bare PMMA fault was also much faster than for the gouge experiments, reaching supershear velocities close to the *P* wave speed of 2,700 m s^−1^. As the rupture reached the eastern fault end, a secondary rupture front propagated back across the fault toward the western end, similar to secondary rupture fronts as observed by, for example, Kammer and McLaskey (2019) and Xu et al. (2019). The rupture velocity of this secondary front was lower than that of the primary rupture, with an average rupture velocity of ~1,200 m s^−1^, close to the Rayleigh wave velocity of 1,237 m s^−1^. Following the secondary front, shear stress increased near the eastern end of the fault until this fault end reruptured, about 0.4 ms after the main event. Such a shear stress increase and rerupture also was observed 0.2 ms later near the western end, and then for the second time at the eastern fault end. Rerupturing events also occurred at supershear rupture velocities. Compared to the gypsum fault experiment for which only sub‐Rayleigh rupture velocities in the range 0.2–0.7 *V*_*R*_ were observed, the rupture velocities were much faster on the bare PMMA fault, with supershear rupture velocities on the different parts of the fault (Figure 5b). Note that determining *v*_*r*_ from the peak stress is subject to some uncertainty, which can explain some rupture velocities falling just above *V*_*P*_.

#### 3.2.2 Fault Stress Distribution on Gouge‐Filled Faults and Bare PMMA Fault

**Figure 6:**
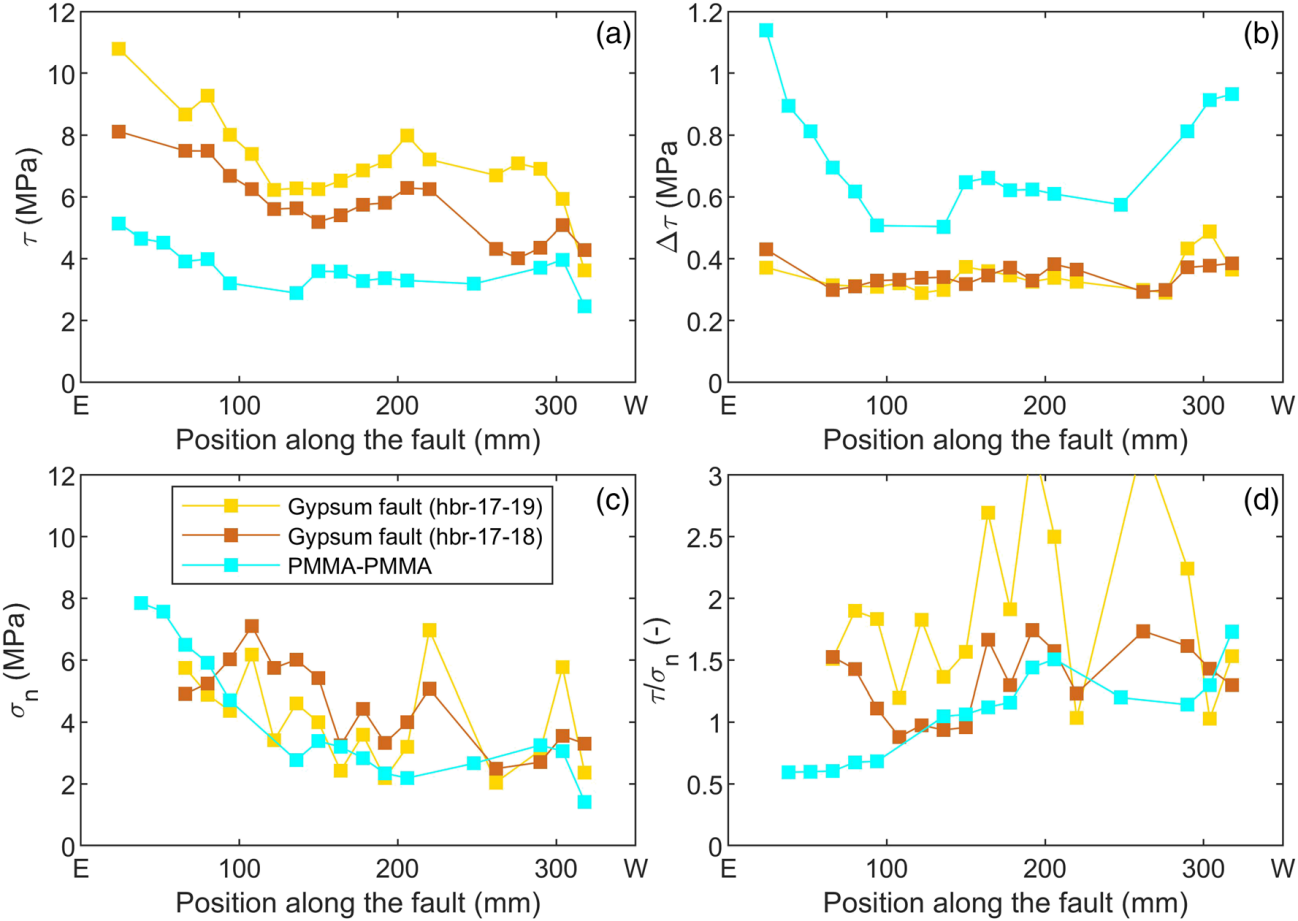
Stresses, stress drops, and stress ratio measured along the gouge‐filled faults and bare PMMA fault. The shear and normal stress are the values recorded prior to stick‐slip at *σ*_2_ = 5 MPa and *v*_1_ = 1 μm s^−1^. (a) Shear stress *τ*, (b) shear stress drop Δ*τ*, (c) normal stress *σ*_*n*_, and (d) the ratio between shear and normal stress *τ*/*σ*_*n*_.

Figure 6 shows the shear stress and the normal stress distributions prior to the main stick‐slip events observed in Figure 4, as well as the stress drop observed during the event. Note that for each experiment these stress distributions are persistent for the entire slip stage at 5 MPa and 1 μm s^−1^. For PMMA the magnitude of recorded stresses also does not change significantly between events, but for the gouge‐filled faults the local shear stresses increases by 0.5–1 MPa from the beginning to the end of the slip stage, whereas the local normal stresses decrease by 1 MPa (not shown). Shear stresses are lowest for the PMMA fault, reflecting the lower friction coefficient that was also observed macroscopically. The average normal stress of 5 MPa recorded for the gouge‐filled faults (Figure 6c) is significantly less than the macroscopic value (*σ*_*n*_ * = 10 MPa). Note however that normal stress measurements near the eastern fault end are not available. As for the PMMA fault, the normal stress near this fault end may be concentrated, as also supported by the fault normal displacements derived from DIC measurements (next section). In this case the average normal stress along would be higher, underlining the importance of having good spatial resolution when comparing local fault stress to macroscopic, far‐field values. An additional explanation for the discrepancy between the recorded normal stresses and the macroscopic value could be the loss of gouge from, or the presence of less compacted gouge along the upper and lower fault margins. Visual inspection of the samples after the experiments suggested that the gouge layer up to a few millimeters from the fault ends and upper and lower fault margins was indeed less compacted. FE model results show that gouge loss or the presence of less compacted gouge along the faults bottom margin will lead to reduced normal stresses on the bottom surface of the PMMA block compared to the normal stress in the center of the fault (Supporting Information S5). The effect of gouge loss may be reflected by the aforementioned decrease of 1 MPa in normal stress with displacement during the slip stage at 5 MPa and 1 μm s^−1^.

**Figure 7:**
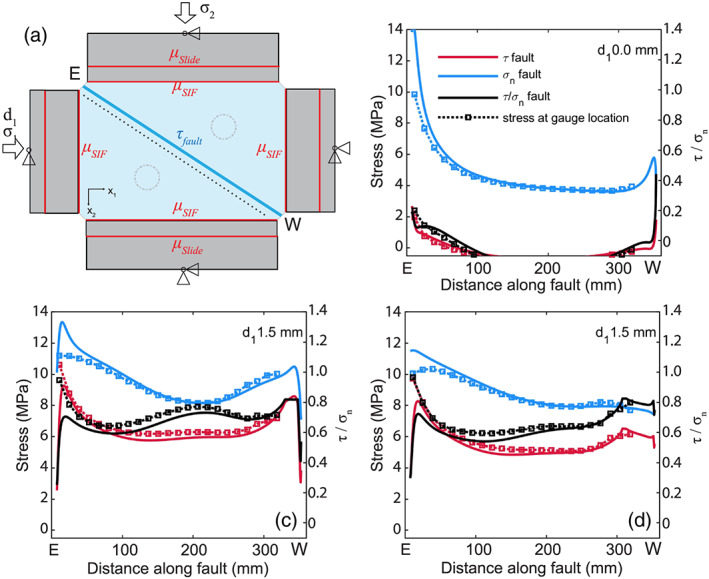
Finite element model of fault stresses. (a) Top view showing the experimental geometry (plane stress) and the imposed boundary conditions. Stresses *σ*_1_ and *σ*_2_ indicate the load‐controlled boundary conditions, and *d*_1_ displacement‐controlled boundary conditions. Triangles indicate supports preventing translation in either *x*_1_ and/or *x*_2_, while allowing rotation. Frictional interfaces included in the model are the fault, the interface between the steel spacers, and the slide bearing plates. (b) Modeled stresses after hydrostatic loading to *σ*_1_ = *σ*_2_ = 5 MPa using a stiff fault (fault normal and shear stiffness 1,000 GPa). (c) Modeled stresses after hydrostatic loading and imposed load point displacement of 1.5 mm using a stiff fault. (d) Modeled stresses after hydrostatic loading and imposed load pointdisplacement using a fault, which is compliant in the normal direction (normal stiffness 10 GPa). Modeled stresses on the fault (solid lines) and at the gauge locations (markers). See for further details on the model the Supporting Information S3.

An asymmetric shear and normal stress distribution was observed for all three experiments, with both shear and normal stress increasing toward the eastern end of the fault (i.e., the end where load was applied, Figure 1). In particular, for the bare PMMA fault the normal stress was very high at the east end compared to the west end. This asymmetric trend could be reproduced qualitatively by FE modeling (Figure 7). The asymmetry of the modeled stress derived from the boundary conditions, mainly the friction of the PMMA‐steel interface and the slide‐bearing plates (Supporting Information S4). Note that the modeled stress distribution was also sensitive to deformation of the fault zone itself, which is particularly important for the experiments with a gouge‐filled fault because the gouge layer may compact. A more compliant fault zone in the shear and/or normal direction relaxed respectively the peaks in shear and/or normal stress (e.g., Figure 7d). Hence, compaction of the gouge‐filled layer might locally reduce normal stress concentrations with respect to those on the PMMA fault, such as stress concentrations at the fault ends; however, it was not possible to place strain gauges very close to the fault ends to confirm this.

As the normal stress on the bare PMMA fault decreased toward the west end of the fault, the modeled *τ/σ*_*n*_ was higher on the western half of the fault, as also observed from the strain data. For the bare PMMA the W‐end is the side where most precursory slip was observed (Figure 4e) and where fault rupture nucleated persistently, from the edge of the slow slip zone near SG248 (e.g., Figure 4f). Although the overall distribution of fault stress is similar for the gouge‐filled fault, smaller length scale stress variations were observed, which were lacking for the bare fault (Figure 6). This is in particular visible in the normal stress distribution, with for example strong normal stress concentrations near SG220 and SG306 (Figure 6c). Stress variability was larger for hbr‐17‐19 than for hbr‐17‐18. On average *τ/σ*_*n*_ was larger on the western side of the fault, and lowest between 50 and 150 mm from the eastern end. The small length scale stress variations were not reproduced in the FE model but appear to derive from the presence of the gouge layer. In the next section we further analyze the deformation of the gouge layer itself.

#### 3.2.3 Fault Relative Shear and Normal Displacements During Loading and *σ*_2_ = 5 MPa

**Figure 8:**
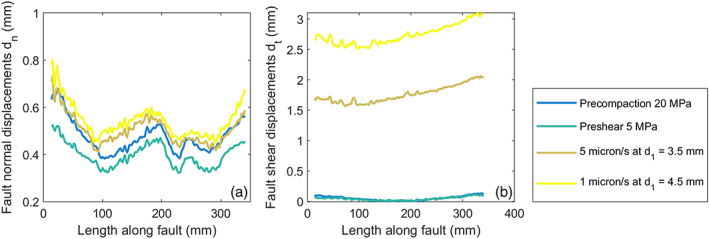
Fault normal and shear displacements along the fault obtained from DIC measurements, at various stages during the experiment. The along‐fault distance is given with respect to the eastern end of the fault. Displacements are relative to the start of the experiment when the sample was not loaded. (a) Fault normal displacements. (b) Fault shear displacements.

The fault gouge not only accommodates shear displacement, but can also accommodate fault normal deformation—that is, the gouge layer can experience compaction. Digital Image Correlation showed that during the precompaction phase (*σ*_1_ = *σ*_2_ = 20 MPa), the average fault‐normal displacement *d*_*n*_ (hereafter: compaction) over the fault was almost 0.5 mm, which is 25% of the original thickness of the gouge (Figure 8a). However, compaction was very heterogeneous along the fault, with maxima at the two fault ends and at the center, the largest of which was found near the eastern end of (*d*_*n*_ = 0.65 mm) where the FE model also predicted the highest normal stress (Figure 7). A smaller scale heterogeneity in compaction was observed at 250 mm from the eastern fault end, where a local maximum was flanked by two minima at 220 and 270 mm from the eastern fault end. At the start of shearing (*σ*_2_ = 5 MPa) the same distribution was still present on the fault (note that the difference in *d*_*n*_ at 20 MPa and preshear 5 MPa is due to elastic relaxation of the PMMA to the sides of the fault). Even after 4 mm of load point displacement the compaction pattern persisted.

Shear displacement *d*_*t*_ was distributed more homogeneously along the fault. Prior to shearing, the measured shear displacement was zero except at the ends. Here, the different shape of the forcing blocks (the corner of one block has an angle of 33.7, the other of 56.3°) and the proximity to the steel forcing blocks causes differential shear deformation across the fault (see also Figure 7a). During shearing the shear displacement was largest at the western end (consistent with the higher shear stress) and decreased toward the other end, reaching a local minimum 80 mm from the end. At a load point displacement *d*_1_ of 4 mm, the average local *d*_*t*_ was 2.75 mm, showing that the PMMA blocks accommodate more than 1 mm of the imposed displacement elastically.

### 3.3 Nucleation on the Gypsum Fault at Lower Confining Stresses

**Figure 9:**
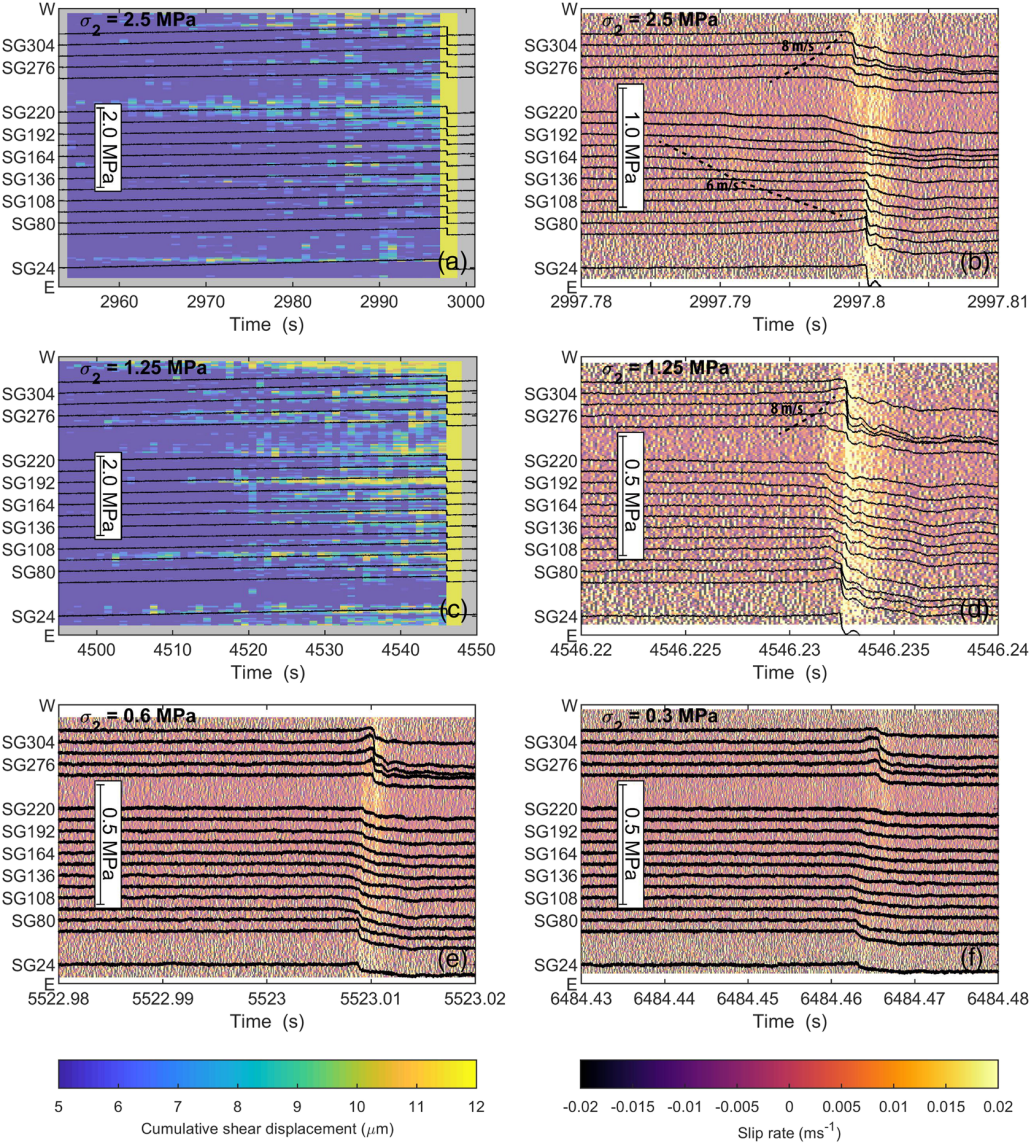
Comparison of local shear stress changes and slip during slip events on the gypsum fault (experiments hbr‐17‐19) at different *σ*_2_. Background colors give cumulative shear slip or the slip rate obtained from Digital Image Correlation. Note that the cumulative shear slip is truncated at 15 μm to highlight slip occurring prior to the slip events. (a) Slip event at *σ*_2_ = 2.5 MPa, (b) zoomed‐in view of the slip event in (a). Dashed lines show the propagation velocity of the nucleation zone, (c) slip event at *σ*_2_ = 1.2 MPa, (b) zoomed‐in view of the slip event in (c). Dashed lines show the propagation velocity of the nucleation zone. (e) Slip event at *σ*_2_ = 0.6 MPa; (f) slip event at *σ*_2_ = 0.3 MPa.

With lower normal stress precursory slip became more widespread and was observed over a larger part of the fault prior to nucleation of instability. The image data for *σ*_2_ = 2.5 MPa show the occurrence of precursory slip in the center of the fault (SG192–SG220) and near the western fault end (Figure 9a), accumulating to ~10 μm prior to the onset of instability. At *σ*_2_ = 1.2 MPa more pervasive precursory slip was observed over the fault, prominently near the western fault end but also in the fault center and the eastern fault end (Figure 9c). At the lowest *σ*_2_ of 0.6 and 0.3 MPa precursory slip became more pervasive, an rupture nucleated as the slow slip zone triggered the eastern end of the fault.

Nucleation occurred persistently from SG220, about two thirds from the eastern fault end. Precursory slip can be observed in this region over almost the entire interseismic period, as well as near the western end (e.g., Figure 9a). The nucleation zone size increased and interacted more with the sample ends (Figure 9). In fact, no constant rupture velocity was attained before the nucleation zone reached the ends, indicating that the nucleation process was ongoing and the slip zone was still accelerating. The length of the nucleation zone increased with the lower *σ*_2_ to cover most of the fault center. For example, at 1.2 MPa nucleation occurred along the fault center from SG94–SG262, and at the lowest *σ*_2_ of 0.3 MPa slow slip occurred from SG94–SG220 (Figure 9f).

As the slow slip zone accelerated from the fault center, shear stress was transferred to the fault ends until one of the fault ends ruptured and rupture backpropagated along the fault—that is, a secondary rupture event. This secondary rupture either propagated back across the entire fault, or was arrested and followed by another rupture event. An example of the first kind of event is shown in Figure 9d (*σ*_2_ = 1.25 MPa). Here, slow yielding was observed in the center part of the fault (SG136–SG220) until at 4546.232 s this slow slip zone accelerated and propagated toward the fault ends. Rupture then started at the east end of the fault and propagated across the fault toward the western fault end (i.e., secondary rupture) at a rupture velocity of 540 m^−1^. The secondary rupture resulted in a small additional stress drop in the center, and ruptured the west end of the fault, which had not yet ruptured. In other cases the secondary rupture was arrested or strongly decelerated in the fault center, and the other fault end ruptured in a separate event (e.g., Figures 9b, 9e, and 9f). At *σ*_2_ = 2.5 MPa for example, first the west end ruptured, but this slip event was then arrested in the fault center (Figure 9b). About 1 ms later, rupture occurred at the east end of the fault, propagating back across the fault at a rupture velocity of ~300 m s^−1^, resulting in a second, slower slip event at the west end. At lower *σ*_2_ the slow slip nucleation zone covered a large part of the fault, first rupturing the east end of the fault. Rupture of the east end then triggered rupture of the west end 2–3 ms later.

**Figure 10:**
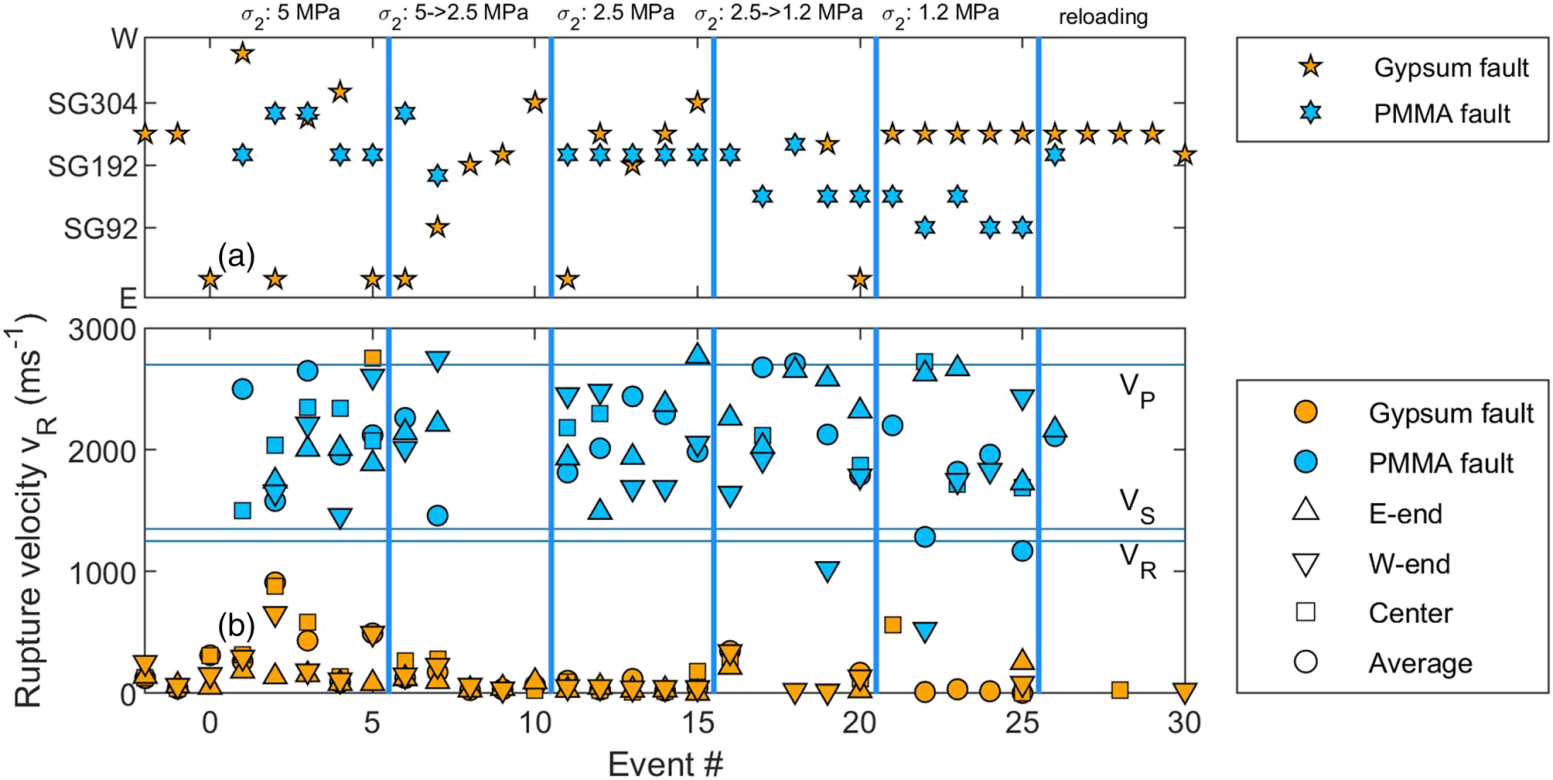
Nucleation site and rupture velocities measured on the gypsum gouge faults and the bare PMMA fault at lower confining stresses. (a) Nucleation site for the different faults given as distance along the fault (sensor locations as in Figure 3). The nucleation site is taken as the strain gauge location at which slow yielding was first observed, or in case no such slow slip zone is recognized, the point from which rupture accelerates. (b) Rupture velocities measured by fitting peak shear stresses during rupture. The average rupture velocity (squares) is the rupture velocity measured after rupture propagates at a constant velocity, or, for events nucleating from the center, the average velocity with which the slip zone propagates toward the fault ends. The rupture velocity is also determined for the east end, west end, and center, where the rupture velocity is averaged between respectively SG24–SG122, SG248–SG318, and SG136–SG220.

The largest stress drop was typically observed near the western fault end, and the smallest stress drop in the fault center, consistent with the stress drop distribution seen at *σ*_2_ = 5 MPaa (Figure 6b). Toward lower *σ*_2_ the local stress drops decreased as also observed in the macroscopic data (Figure 2). Although the stress drops became smaller, the duration of the stress drop increased, which was reflected by the lower slip rates computed using DIC (Figure 9b vs. 9e). The velocity with which the nucleation zone propagated toward the fault ends also decreased with lower *σ*_2_ (Figure 10b), with, for example, velocities of 10–20 m s^−1^ at *σ*_2_ = 1.2 MPa. For comparison, rupture velocities on the PMMA fault remained supershear, although locally several sub‐Rayleigh velocities were observed near the west end and in the fault center at *σ*_2_ = 1.2 MPa (Figure 10b).

#### 3.3.1 Events During Unloading and Reloading

As *σ*_2_ was lowered during the experiment (while the load point position of the press in the *x*_1_ direction was kept constant), the normal stress and shear stress decreased. During these unloading stages several slip events were recorded. For these events, yielding on the gouge‐filled fault started from SG220 or from the east end of the fault (Figure 10a). For the PMMA fault, the nucleation site shifted gradually from SG192‐SG248 at the highest *σ*_2_ to SG94‐SG136 at the *σ*_2_ = 1.2 MPa or lower. Upon increasing the confining stress to 5 MPa, the nucleation site shifted back to the same location as during the first loading stages.

## 4 Discussion

The experiments presented in this study are the first where rupture nucleation was observed closely along a fault filled with gouge, sandwiched by compliant PMMA forcing blocks. For reference, also a bare PMMA fault was deformed. The main differences observed for the gouge‐filled fault with respect to the bare PMMA fault were a more variable location of nucleation, a more heterogeneous fault stress, more precursory slip, and lower rupture velocities. Here we discuss the nucleation process in relation to the observed and modeled fault stresses and theory, compare our results to other large‐scale experiments on bare rock or polymers, and relate the observed nucleation to theory.

### 4.1 Fault Stress and Location of Rupture Nucleation

The state of stress on the fault is important for understanding rupture nucleation and propagation. Strain measurements along the fault zone indicate that the shear and normal stress are heterogeneous along the fault, with stronger small length scale stress heterogeneities observed for the gouge‐filled faults compared to the bare PMMA fault (Figure 6). The stress heterogeneities derive from the experimental boundary conditions, as well as from the interface and/or gouge properties. In the following, we compare stress distributions observed in other experimental setups to the stresses observed in this study and investigate the effect of the boundary conditions and strain measurement location.

#### 4.1.1 Previous Studies on the Effect of Experimental Boundary Conditions on Fault Stress, and Its Relation With Nucleation

It is commonly observed that experimental boundary conditions cause a heterogeneous shear and normal stress distribution along experimental faults. Strain gauge measurements in direct shear experiments show that shear stress concentrations develop near one or both fault ends, depending on the sample geometry and loading configuration (e.g., Bayart et al., 2016; Ben‐David et al., 2010; Langer et al., 2013; Xu et al., 2018; Yamashita et al., 2018). For example, applying an along‐fault displacement to the end of one of the forcing blocks while allowing along‐fault displacement on the opposite end of the second forcing block results in a shear stress concentration near one end (at the side at which the load is applied), and a stress low near the other end (Bayart et al., 2016; Ben‐David et al., 2010). When shear displacement on the opposite end of the second forcing block is resisted, shear stress concentrations develop near both ends (Bayart et al., 2016; Xu et al., 2018; Yamashita et al., 2018). Similarly, normal stress concentrations in the direct shear experiments form at one or both ends, as observed from strain gauge measurements (Bayart et al., 2016; Ben‐David et al., 2010), pronounced fault wear (Mclaskey & Yamashita, 2017), pressure sensitive films (Yamashita et al., 2018), and modeling (Kammer et al., 2015). The distribution of the shear and normal stresses along the experimental faults is furthermore affected by a other geometrical factors and boundary conditions, such as the sizes of, for example, the (PMMA) forcing blocks relative to each other, whether shear loading is applied uniformly or at a point (Bayart et al., 2016), the location at which the shear load is applied (Ben‐David et al., 2010), deformation of the frame containing the experimental setup (Ke et al., 2018), and whether motion is allowed between hydraulic presses and the forcing blocks (Guérin‐Marthe et al., 2019; Langer et al., 2013). Depending on the relative magnitude of the shear and normal stresses, maxima in *τ/σ*_*n*_ will form at some distance from the fault end(s) (e.g., Guérin‐Marthe et al., 2019; Yamashita et al., 2018). The stiffness of the forcing block and the surrounding are also important factors that affect the fault stress. The large stiffness contract between polymers and their surroundings may cause high stresses predominantly at the boundaries, whereas the smaller stiffness contrast in experiments using granite or gabbro forcing blocks result in stress maxima further to the center of the fault (Ke et al., 2018). For constant fault friction rupture is expected to start at locations where the ratio *τ/σ*_*n*_ is largest (as observed by, e.g., Ben‐David et al., 2010; Ke et al., 2018), though sometimes rupture nucleation occurs from a lower stress area (Yamashita et al., 2018). Note that rupture nucleation may be complex as for example multiple slow slip zones can coalesce to generate instability (Fukuyama et al., 2016), which may not necessarily be at a maximum in *τ/σ*_*n*_. Also, increased loading rates can cause a more heterogeneous stress state and more variability in the position of nucleation (Xu et al., 2018).

#### 4.1.2 Fault Stresses and Nucleation on the Bare PMMA Fault

In the biaxially loaded saw cut setup as used in this study the PMMA forcing blocks are supported on all sides, with loads being applied on two sides, and load resisted on the other two sides (Figure 1a). The stress distribution that was measured was asymmetric with higher shear and in particular high normal stresses on the eastern side of the fault (Figures 6a and 6c), which is the side from which loading is applied (Figure 1a). This trend could be explained (qualitatively) with the FE model (Figure 7 and Supporting Information S4), which shows high normal stresses and low *τ/σ*_*n*_ on the east end of the fault. Conversely, the observed and modeled normal stress on the western side of the fault was low and the stress ratio *τ/σ*_*n*_ was high (Figures 6c, 7c, and 7d). Though the modeled stresses fit well with the observation, our modeling study also shows that the stress on the fault is highly sensitive to the boundary conditions. In addition to the factors mentioned in the previous section (configuration and relative size of the forcing blocks, location at which and area over which the loads are applied or resisted, the stiffness of the forcing blocks, and the surroundings), the FE results show that fault stress is affected significantly by friction between PMMA and the steel spacers, friction of the slide‐bearing plate, and the degrees of freedom of the steel spacers. For the current setup the asymmetry in normal stress derived mainly from friction between PMMA and the steel boundaries, which was measured in a separate experiment to be 0.3–0.4. Knowledge of the effect of the boundary conditions on the fault stress is important; it not only allows one to predict or explain nucleation location but also allows one to predict the arrest locations of rupture (e.g., Kammer & McLaskey, 2019; Ke et al., 2018), or to manipulate the rupture nucleation and velocities by, for example, changing the location of the applied loads (Bayart et al., 2016).

Nucleation on the PMMA fault occurred persistently at about 2/3 to 3/4 of the fault length (SG192–SG248, Figure 5a), which is at the border of the precursory slip zone near the western fault end (Figure 4e). The precursory slip zone and nucleation location coincides with a region of high *τ/σ*_*n*_ in the observed and modeled stresses (Figures 6d and 7c). No clear nucleation zone was observed in the strain gauge data, although DIC does show precursory slip occurring near the western end of the fault, close to the nucleation site. The rate‐and‐state parameters of PMMA‐PMMA are not known, but the nucleation length observed on PMMA faults in other studies at a lower normal stress of ~5 MPa appears to be in the order of a centimeter or less (Ben‐David et al., 2010; Svetlizky & Fineberg, 2014), which would not be resolved well by the strain gauge array.

#### 4.1.3 Fault Stresses and Nucleation on the Gouge‐Filled Fault

**Figure 11:**
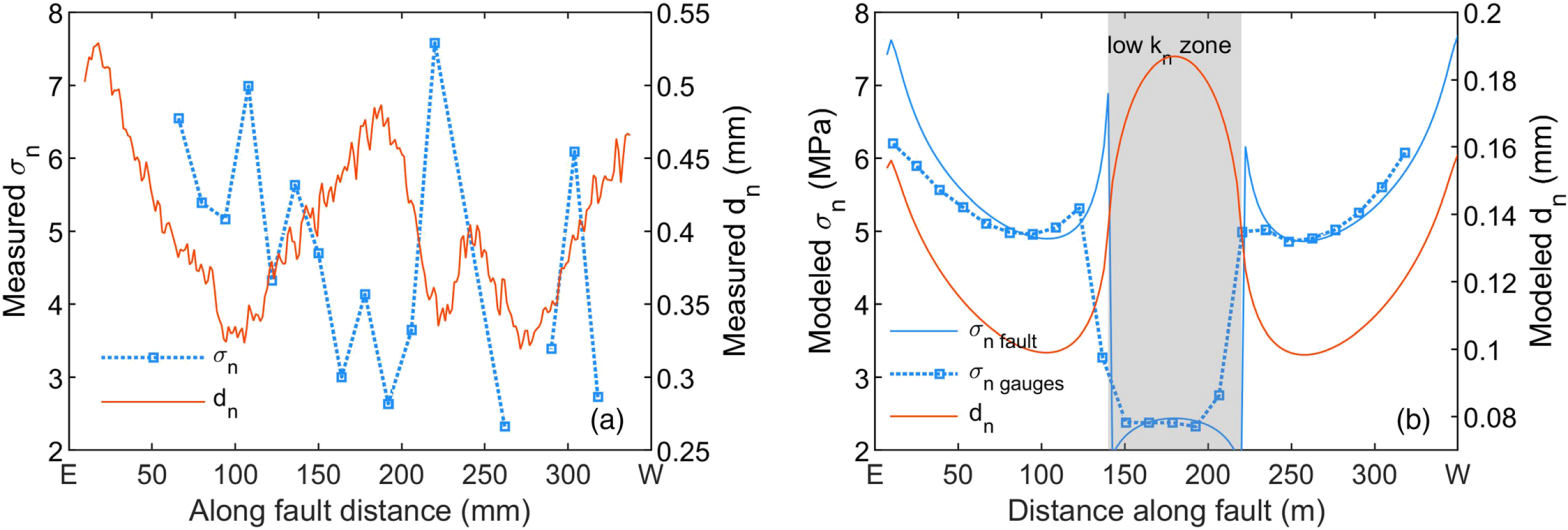
Relation between normal stress and fault normal displacement after precompaction. (a) Normal stress recorded by the strain gauges and fault normal displacement *d*_*n*_ obtained from DIC. (b) Modeling example of the fault‐normal displacement and normal stress distribution for a fault with heterogeneous fault‐normal properties. The fault‐normal stiffness *k*_*n*_ of the center segment is 4 times lower than in the surrounding segments, causing increasing elastic fault normal deformation.

Nucleation on the gouge‐filled fault at *σ*_2_ = 5 MPa was more variable than on the bare PMMA fault, with nucleation occurring at about two thirds of the fault length as for the PMMA fault (Figure 5a) and also from both fault ends. A number of events nucleating at the fault ends were arrested before transitioning into a dynamic event. Some of these foreshocks at the western end triggered slip in the middle of the fault, eventually resulting in rupture of the entire fault (e.g., S1‐7, Figure 3). Multiple interacting slow slip zones thus led to nucleation of unstable sliding; coalescence of slow slip zone has also been recognized as a mechanisms for instability in numerical simulations (Kaneko & Ampuero, 2011). The different slip zones may be related to the heterogeneous stress (in particular the normal stress) that was observed on the western half of the gouge‐filled fault (Figure 6). The normal stress heterogeneities correlate to the heterogeneous fault compaction observed after the precompaction phase and during shearing (Figure 11a), with high normal stresses corresponding to regions experiencing relatively little compaction (Figure 11a). Such a region may form an asperity. Heterogeneous compaction could result from the (inevitably) uneven distribution of the amount of gouge material along the fault and/or locally heterogeneous gouge porosity. A simplified model setup incorporating a central segment with a lower fault normal stiffness (to simulate fault compaction) shows how fault segments that experience increased compaction support less normal stress, thus resulting in a higher *τ/σ*_*n*_ (Figure 11b). Normal stress is transferred to adjacent fault segments as observed at 100 and 205 mm (Figure 11a), which in turn would have a low *τ/σ*_*n*_. This is comparable to stress concentrations predicted by the anticrack model (or contractile Eshelby inclusion) (e.g., Sternlof et al., 2005). To some extent the heterogeneous compaction is comparable to bare rock or polymer experiments, where asperities sustain high normal stresses (Selvadurai & Glaser, 2015) and grooves form regions of low normal stress (Yamashita et al., 2018). However, the magnitude of the fault‐normal deformation of the gouge layer may be much larger than the typical amplitude of the asperities, depending on the roughness of the bare rock or polymer fault. The variations in normal stress along the (center of the) gouge‐filled fault are more than twice as large as variations in normal stress measured along smooth (1–3 μm roughness) bare polymer surfaces under comparable loading conditions (e.g., Bayart et al., 2016; Ben‐David et al., 2010), showing how the use of gouge can create additional stress heterogeneity. Sliding on rougher bare rock fault may however cause more comparable fluctuations in normal stress on short length scales (Yamashita et al., 2018). Note that the displacement on the fault in the current experiment is limited; heterogeneities arising from compaction might be smoothed out as the imposed shear displacement exceeds their length scale.

On the gouge‐filled fault ruptures also nucleated from both fault ends, which was not seen on the PMMA fault. On the PMMA fault, normal stress near the fault ends is likely concentrated, in particular near the E‐end of the fault (Figure 6c). For the gouge‐filled fault compaction of the gouge can locally reduce the normal stress, as also discussed in the previous paragraph. Significant gouge compaction near both fault ends was observed from DIC (Figure 8); this could have led to a lower normal stress, higher *τ/σ*_*n*_ and nucleation near the fault ends compared to the bare PMMA fault.

Note that if the length scale of the stress variations is much larger or much smaller than the critical nucleation length, the nucleation process may be less affected (Ray & Viesca, 2019). This will affect nucleation at lower *σ*_2_ where the nucleation length becomes larger. The nucleation length scales observed in the experiments can be compared to theoretical estimates (section 1.1) calculated from the rate‐and‐state parameters of gypsum and stiffness of the PMMA blocks (Supporting Information S2). For a normal stress of 10 MPa (*σ*_2_ = 5 MPa) and load point velocities closest to those used in the current study, the lower limit nucleation half‐length *L*_*b*_ ranges from 0.01–0.05 m (Figure S4a), and *L*_*∞*_ ranges from 0.04–0.3 m (Figure S4c). Modeling and experiments show the nucleation process is divided into a stable, quasi‐static phase (rupture velocities often in the order of cm s^−1^ to m s^−1^), an accelerating phase during which rupture accelerates to the third phase, dynamic rupture propagation (e.g., Kaneko et al., 2016; Ohnaka & Shen, 1999). Theoretical estimates of the critical nucleation length correspond to the transition from the quasi‐static phase to the accelerating phase (here defined as 0.05 *V*_*R*_). At *σ*_2_ = 5 MPa no clear quasi‐static nucleation zone could be recognized from the strain data, though image data shows precursory slip over a several parts of the fault throughout the interseismic period (Figure 4a). In some cases rupture started from the fault end and propagated at a steady velocity of 200–400 m s^−1^ (Figure 3c), without any clear yielding at the fault end where nucleation started. In other cases rupture was still accelerating toward the fault ends at propagation velocities of 0.1–0.2 *V*_*R*_, but here also no clear quasi‐static nucleation zone was observed. The nucleation zone size may be too small to be well resolved by the strain gauges (i.e., <0.02 m), which would be on the lower end of the theoretical estimates. Several factors may cause a reduction of the nucleation length near the fault ends, such as the local high normal stresses due to heterogeneous compaction or higher normal stresses and/or the high shear stress rates at the fault ends (e.g., Guérin‐Marthe et al., 2019). The stress intensity factor at the tip of a slip zone originating from a free edge is larger than the stress intensity factor at the tips of an embedded slip zone (Ke et al., 2018; Tada et al., 2000), which could result in small nucleation lengths near the fault ends. Also, if the gouge layer has a lower stiffness it could reduce the nucleation length, if the thickness of the gouge layer is larger than or a significant fraction of the critical nucleation length (Kaneko et al., 2011). The gouge thickness of ~1.2 mm may indeed be a nonnegligible part of the critical nucleation length for a gouge with a lower stiffness.

At lower *σ*_2_ the nucleation length increased visibly. The nucleation zone always started from two thirds along the fault, and the expanding nucleation zone reached the sample ends, while it was still accelerating, similar to what is also observed in bare rock experiments (e.g., Fukuyama et al., 2016; McLaskey & Kilgore, 2013). At *σ*_2_ = 2.5 MPa was ~0.2–0.25 m (see Figure 9b), which is in agreement with theoretical estimates mentioned which for *σ*_2_ = 2.5 MPa become 2 times as large as the values mentioned. No nucleation from the fault ends was observed; the length scale of the stress concentrations near the fault ends was much smaller than the theoretical nucleation length. It is possible that precursory slip occurs near (predominantly the western) fault end and triggers rupture nucleation at two thirds of the fault by merging with another slow slip zone in the central parts. In general the stresses *τ/σ*_*n*_ are larger in the western half of the fault, favoring nucleation of slip (Figure 6). For the lowest *σ*_2_ of 0.6 and 0.3 the theoretical estimates of the nucleation length are almost all larger than the fault length. Instability still occurred after the precursory slip expanded from the central parts of the faults toward the eastern end where the normal stress was higher. The expanding nucleation zone then triggered the eastern end, upon which rupture propagated back over the whole fault. The widespread precursory slip at the lowest stresses is consistent with numerical modeling which shows that velocity weakening patches with lengths just larger than the critical nucleation length accommodate significant aseismic slip (e.g., Chen & Lapusta, 2009). Note however that in this case slip on the patches is driven by creep in the surrounding material, which is different from our fault where the ends are free; it remains to be determined whether similar trends would be observed for free fault ends versus a creeping matrix.

#### 4.1.4 Effect of Loading Conditions

In the experimental setup used in this study both the shear and normal stress increase as the sample is loaded. This is similar to for example experiments by Latour et al. (2013), but different from other many other experimental setups where, for example, shear stress is increased while keeping the normal stress constant, in a saw cut setup (e.g., Kato et al., 1992; McLaskey & Kilgore, 2013) or in a direct shear setup (Fukuyama et al., 2018; Mclaskey & Yamashita, 2017, Yamashita et al., 2015). The different loading conditions lead to a different state of stress on the fault (section 3.2.2), but the nonconstant normal stress can also affect the nucleation process and the location of nucleation. A modeling study by Kaneko et al. (2016) shows more variability in nucleation characteristics under increasing shear and normal stress, compared to only increasing the shear stress. Reduction of normal stress while keeping the shear stress fixed led to more variability in nucleation locations along a PMMA fault (Shlomai & Fineberg, 2016). In our experiment the confining stress *σ*_2_ was decreased several times—that is, unloading. During unloading the normal stress decreased, but the shear stress increased triggering several slip events which reduced the shear stress. In light of the importance of the loading conditions it is interesting to compare these unloading events were compared to the events during loading. For the gouge‐filled fault the nucleation location did indeed show a wider distribution (Figure 10a). This could be related to the heterogeneous fault stress. Note however that the loading rate during unloading is larger, which may be another explanation for the larger variability (e.g., Kaneko et al., 2016; Xu et al., 2018). More research is required to investigate the relative effects of the (un)loading conditions, loading rate, and the heterogeneous fault stresses. For the bare PMMA fault the nucleation site shifted from a position approximately two thirds of the fault length to a position at one third of the fault length (Figure 10a). This appeared however to be correlated with the decrease of *σ*_2_ in general, not just unloading. At lower stresses, the stress maximum predicted by the FE model on the western side of the fault becomes flatter, which may be one explanation for the shift in nucleation location (as well as for the reverse happening when increasing the stresses).

### 4.2 Effect of the Gouge Layer on Rupture Velocity and Secondary Rupture Fronts

Another difference between rupture on the bare PMMA fault and on the gouge‐filled faults was the rupture velocity (Figure 5b). The rupture front of the primary ruptures on the bare PMMA fault reached supershear velocities, but the rupture velocity observed for rupture events on the gouge‐filled faults was lower. Note that although the number of slip events that was analyzed was relatively small, quite some variability in (average or local) rupture velocity was observed with average rupture velocities of 0.1–0.7 *V*_*R*_, the Rayleigh wave speed of the PMMA forcing blocks. In only one event did the rupture velocity locally exceed *V*_*R*_ (Figure 4b)_._ The rupture velocities on the gypsum fault are consistent with but in the lower range of rupture velocities seen in other large‐scale experiments on bare polymer faults at similar normal stress (e.g., Ben‐David et al., 2010).

At the highest confining stress (*σ*_2_ = 5 MPa) ruptures nucleating at either one of the fault ends typically attained an average rupture velocity 0.2–0.3 *V*_*R,*_ (Figures 3c and 5b) with one event attaining an average rupture velocity of almost 0.7 *V*_*R*_ (Figures 3d and 5b). Ruptures that nucleated closer to the center propagated at lower velocities (<0.1 *V*_*R*_), but these ruptures were still accelerating as rupture reached the fault ends (e.g., Figure 3e)—that is, the critical nucleation length had not been exceed. Similarly low rupture velocities were observed during the nucleation phase of rupture on granite (Kato et al., 1992; McLaskey & Kilgore, 2013; McLaskey et al., 2015) and PMMA (Svetlizky & Fineberg, 2014) faults. As the accelerating rupture reached the fault ends, a secondary rupture front backpropagated over the fault with a rupture velocity of 0.2–0.5 *V*_*R*_ (Figure 3d), in the same range as the rupture velocities of primary rupture nucleating at the fault ends. The velocities of the secondary fronts on the gouge‐filled faults are lower than the rupture velocities typically observed for secondary ruptures occurring in bare rock experiments using granite (Kammer & McLaskey, 2019) or gabbro (Xu et al., 2019) forcing blocks, which are in the range of 0.85–0.95 *V*_*R*_. Secondary ruptures were also observed on the bare PMMA fault, as the rupture propagating from the west end reached the east end; these secondary fronts did have a rupture velocity close to the Rayleigh wave speed (Figure 4f). Note that in particular for the gouge‐filled fault the secondary wave front was only weakly visible in the strains recorded along the fault, compared to secondary rupture events on granite or gabbro faults (Kammer & McLaskey, 2019; Xu et al., 2019). This could be due to the resolution limit of the strain gauges used in this study (see section 2.3). The secondary ruptures detected on granite faults (Kammer & McLaskey, 2019; Xu et al., 2019) and primary ruptures on PMMA (Svetlizky & Fineberg, 2014) have a typical wavelength of 10–30 mm. If the wavelength of the secondary ruptures in this study is similar, for the strain gauge size of 5 mm used here it may not be well resolved, in particular, if there is no stress drop associated with the secondary rupture. Strain measurements may show no stress drop even though a stress drop on the fault occurs, due to the distance between the fault and the location of the strain gauge (Kammer & McLaskey, 2019). The secondary ruptures seen on the gouge‐filled faults were associated with a stress drop (e.g., Figure 3d). The secondary ruptures were also visible as a second slip pulse in the image data (e.g., rerupturing of the west end of the fault for the event in Figure 3d), indicating the fault locks after the primary event and is then rerupturing during the secondary rupture. On the PMMA fault the secondary ruptures are clearer because the amplitude of the stress changes was larger (Figure 4f).

The rupture velocity of both the primary and secondary rupture front on the gouge‐filled faults thus were much lower than on the bare PMMA fault and were on the low side of those seen in other bare rock or bare polymer experiments. There are several factors that affect the rupture velocity, including the local state of stress, the properties of the gouge and the medium surrounding the fault, the loading rate, the size of the fault. A positive correlation was found between local rupture velocity and the (local) ratio *τ/σ*_*n*_ in polymer experiments (Ben‐David et al., 2010) or on smaller‐scale granite faults (Passelègue et al., 2013). The events on the gouge‐filled faults in our study nucleated at a relatively high *τ/σ*_*n*_ near the fault ends, while on the rest of the fault *τ/σ*_*n*_ was lower, which could result in lower rupture velocities (Figure 4, Figure 6). However, for the PMMA fault the stress state was also heterogeneous with lower *τ/σ*_*n*_ toward the east end, but rupture velocities were much faster than for the gouge‐filled faults. Possibly, the smaller wavelength normal stress concentration that were observed along the gouge‐filled fault but were absent along the PMMA fault could have decelerated rupture. Laboratory experiments showed that a higher degree of stress heterogeneity causes more complex rupture behavior and a region with heterogeneous stresses may indeed cause ruptures to decelerate, though ruptures have also been observed to accelerate after overcoming the heterogeneous stress region (Latour et al., 2013). An alternative explanation for the low rupture velocities can be found in the properties of the gouge layer itself. The fault zone is likely a zone of low stiffness (though unfortunately its elastic properties are unknown) and can have a lower Rayleigh wave velocity than PMMA. For bare PMMA ruptures wavelengths in the order of 10–20 mm have been observed for the primary front, which decrease to several mm at high rupture velocities (Svetlizky & Fineberg, 2014). The gouge layer is (after compaction) ~1.2 mm wide and may not be insignificant with respect to the wavelength of the rupture fronts. If its Rayleigh velocity of the gouge is significantly lower than that of PMMA, it may reduce the rupture velocity. Furthermore, deformation of the gouge layer during shearing could attenuate the propagating (secondary) ruptures. Attenuation due to the progressive accumulation of fault gouge was proposed as one of the explanations for the decrease in rupture velocities of secondary rupture front seen on an initially bare gabbro fault (Xu et al., 2019). The decrease in *V*_*R*_ seen on the gabbro fault was small (1.4%), but the accumulated gouge layer had a thickness of 20 μm. In our case the gouge layer is thicker and may cause more attenuation which may also explain why the secondary rupture fronts are only weakly visible the gouge experiments. In addition, the gypsum gouge has different frictional properties compared to the PMMA fault. This is reflected in the nearly twice as high stress drop observed on the PMMA fault (Figure 6b). A positive correlation has been observed in experiments between the stress drop and rupture velocity (e.g., Passelègue et al., 2013), so that faster rupture velocities are expected for the PMMA fault. It is difficult to quantify the relative importance of the different processes on the rupture velocity, but for the gouge it appears that its low stiffness in combination with its attenuating properties and lower stress drop could have resulted in lower rupture velocities than those of the PMMA fault.

### 4.3 Implications for Natural and Induced Seismicity

The experimental results show that spatially heterogeneous stress ratios *τ/σ*_*n*_ affect the nucleation of slow slip in the present laboratory setup. Locally, the fault stress deviates significantly from the far‐field stress. The local stability criterion for rupture nucleation may thus also deviate significantly from the one obtained using far‐field stresses. The transition from stable sliding to stick‐slip can occur at different loading conditions (e.g., different macroscopic normal stress *σ*_n_*) for different experimental setups, depending on the development of stress heterogeneity due to the boundary conditions and heterogeneous compaction of the gouge layer.

The present experiment suggests that fault zone architecture and (variations in) fault gouge composition can be a source of stress heterogeneity within a fault zone. Fault zones cross‐cutting sedimentary reservoirs may have for example a highly variable lithology along the fault plane with phyllosilicate‐rich fault rock and sand lenses (Fredman et al., 2007), and also the degree of cementation may vary along a fault depending on the lithology and amount of brittle deformation it experienced locally (Fisher & Knipe, 1998). Friction experiments on different siliciclastic fault gouge materials found in faults in the Groningen field show how the compaction behavior of these materials can be very different (Hunfeld et al., 2017), which can cause strong normal stress concentrations on fault portions that compact less. Field studies show that exhumed crustal‐scale fault zones are often composed of a phyllosilicate‐rich matrix surrounding competent lenses of other lithologies, forming so‐called fault melanges (Fagereng & Sibson, 2010; Wibberley et al., 2008). During the interseismic period the phyllosilicate‐rich matrix might deform through pressure solution creep and may thus compact more than the competent lenses within the matrix. This could lead to increased normal stresses on the more competent fault lenses, which causes a reduction of the nucleation size and an increased likelihood of unstable sliding when sufficiently loaded by the creeping fault matrix. Whether such an event is able to propagate through the surrounding matrix depends on the spatial distribution of competent fault segments, the frictional properties of both fault constituents and the amplitude and spatial distribution of the stress heterogeneities (e.g., Luo & Ampuero, 2018).

Heterogeneous stresses also form on natural faults subject to tectonic loading, or on faults subject to anthropogenic stress changes. On naturally active faults, and faults on which motion is induced by human activity, geometrical factors (jogs, steps, and seamounts) and fault roughness may produce marked stress concentrations. For anthropogenically induced seismicity, pressure, and or temperature changes may cause additional highly concentrated stresses that cause earthquakes to nucleate, for example, around injection wells (e.g., Galis et al., 2017; Segall & Lu, 2015), around mines, or within producing reservoirs such as Groningen (e.g., Buijze et al., 2019). In the case of producing reservoirs, both shear and normal stress concentrations form as a result of elevated effective stresses and reservoir geometry (e.g., Buijze et al., 2019; Haug et al., 2018; Zbinden et al., 2017). The present experiment underlines that it is important to consider these stress concentrations to understand where slip events start and stop.

## 5 Conclusions

In this study, we investigated rupture nucleation and propagation in an experiment on a 350 mm long gouge‐filled laboratory fault under biaxial loading conditions. The fault was created along the diagonal of a rectangular polymethylmethacrylate (PMMA, or Perspex) block, which was used as forcing blocks because its compliancy reduces the nucleation length scale. The fault zone was filled with a 2 mm thick layer of gypsum gouge, which exhibits velocity weakening and produces slip instabilities at the experimental conditions (i.e., room temperature, normal stresses <12 MPa, and load point displacement rates 1 or 5 μm s^−1^). During the experiment the minimum horizontal stress *σ*_2_ was reduced stepwise from 5 to 0.3 MPa, and then increased again. For reference, a second experiment was performed on a gypsum fault at *σ*_2_ = 5 MPa, as well as an experiment without gouge (bare PMMA fault). Strain gauges and Digital Image Correlation were used to analyze the stress changes and displacements during the stick‐slip cycle. Here we summarize our main findings:

1. Stick‐slip events occurred at all confining stresses investigated. These were audible at the highest confining stresses (1.2–5 MPa) but became inaudible at lower stresses.
2. At the highest confining stress of 5 MPa dynamic events on the gypsum gouge fault nucleated from either one of the fault ends or at approximately two thirds of the fault. In the latter case, nucleation sometimes occurred between two slow slip zones. Rupture nucleation on the PMMA fault consistently started at approximately two thirds of the fault length from the eastern end of the fault, at the edge of the precursory slip zone at the western end. The increased complexity of nucleation on the gouge‐filled fault was related to small length scale normal stress variations which were absent for the bare PMMA fault. These variations derived from variations in compaction of the gouge. Compaction heterogeneity and the related normal stress heterogeneities persisted after at least several mm of sliding on the fault.
3. Stresses obtained from the strain gauge array along the fault margin showed that nucleation sites occurred in areas where the stress ratio (*τ/σ*_*n*_) was high on average. The stress distribution could be reproduced qualitatively by FE modeling. The modeled *τ/σ*_*n*_ distribution was asymmetric, being largest at the western end of the fault, and resulted from the experimental boundary conditions (i.e., the free fault ends, friction between loading pistons and forcing blocks, and rotation of the loading pistons).
4. Nucleation length scales agreed with theoretical estimates using RSF parameters obtained in independent, small‐scale experiments.
5. More precursory slip was observed over a larger area on the gouge‐filled fault than on the bare PMMA fault. Precursory slip occurred near the fault ends and west of the fault center and was correlated to high local stress ratio *τ/σ*_*n*_.
6. Rupture velocities and slip rates were lower on the gouge‐filled faults compared to the bare PMMA fault. Subsonic ruptures were mostly observed for the gouge‐filled fault (0.1–0.7 *V*_*R*_, the Rayleigh wave speed of PMMA), whereas supershear ruptures were observed on the bare PMMA fault. Also, the velocities of secondary ruptures on the gouge‐filled fault were lower (0.2–0.6 *V*_*R*_) compared to those on the PMMA which were close to *V*_*R*_. The lower velocity of the gouge layer or its attenuating properties may have contributed to the lower rupture velocities.
7. At *σ*_2_ = 2.5 MPa rupture nucleation started from approximately two thirds of the fault length, but at lower *σ*_2_ the nucleation zone covered a large area in the center of the fault before reaching the fault end(s). The growth of the nucleation zone was accompanied by several local accelerations in shear stress release until the slip zone approached the fault ends, and the secondary rupture propagated back over the entire fault zone. At these confining stresses the critical nucleation length was close to or larger than the fault length. For the PMMA fault the nucleation length was much smaller.
8. The results illustrate the importance of fault stress distribution in controlling rupture nucleation and propagation, a point that is equally relevant to understanding both natural and induced earthquakes. The nucleation of instability estimated from far‐field stresses may be different from the actual nucleation that results from local (concentrated) stresses on the fault. Local stress lows around the highly stressed nucleation area may “quench” a nucleating rupture, whereas broader regions of high stress promote rupture nucleation and propagation. Fault zone architecture and gouge composition can contribute to strong stress variability within and around a fault zone. Strong normal stress concentrations may develop on fault zone segments or rigid lenses experiencing less compaction.

## Supporting information

Supporting Information S1

## Data Availability

The mechanical data, selected strain data, and selected images can be downloaded from the Yoda repository of Utrecht University (https://public.yoda.uu.nl/geo/UU01/OT6YIY.html), DOI: 10.24416/UU01‐OT6YIY.
