# Supplementary material for: Nucleation of Stick‐Slip Instability Within a Large‐Scale Experimental Fault: Effects of Stress Heterogeneities Due to Loading and Gouge Layer Compaction
Source: J Geophys Res Solid Earth. 2020 Aug 14;125(8):e2019JB018429. doi: 10.1029/2019JB018429 (PMC7507769; doi:10.1029/2019JB018429)
Supplement: Supplementary file 1 — Supporting Information S1 [file JGRB-125-e2019JB018429-s001.docx]

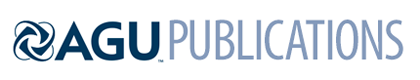


*Journal of Geophysical Research*

Supporting Information for

**Different nucleation characteristics as a result of loading conditions on a gouge-filled large-scale laboratory fault**

L. Buijze^1,2†^, Y. Guo^3^, A.R. Niemeijer^1^, S. Ma^3^ and C.J.Spiers^1^

^1^HPT Laboratory, Department of Earth Sciences, Utrecht University, Utrecht, The Netherlands

^2^ Applied Geosciences, TNO, Utrecht, The Netherlands.

^3^ State Key Laboratory of Earthquake Dynamics, Institute of Geology, China Earthquake Administration, Beijing, China.

**Contents of this file**

Text S1 to S5

Figures S1 to S11

**Additional Supporting Information (Files uploaded separately)**

The mechanical data, selected strain data and selected images can be downloaded from the Yoda repository of Utrecht University <https://public.yoda.uu.nl/geo/UU01/OT6YIY.html>, DOI: 10.24416/UU01-OT6YIY

**Introduction**

In this Supporting Information document we provide additional background to the experimental and numerical results described in the main document.

1. Friction of gypsum gouge measured in triaxial experiments


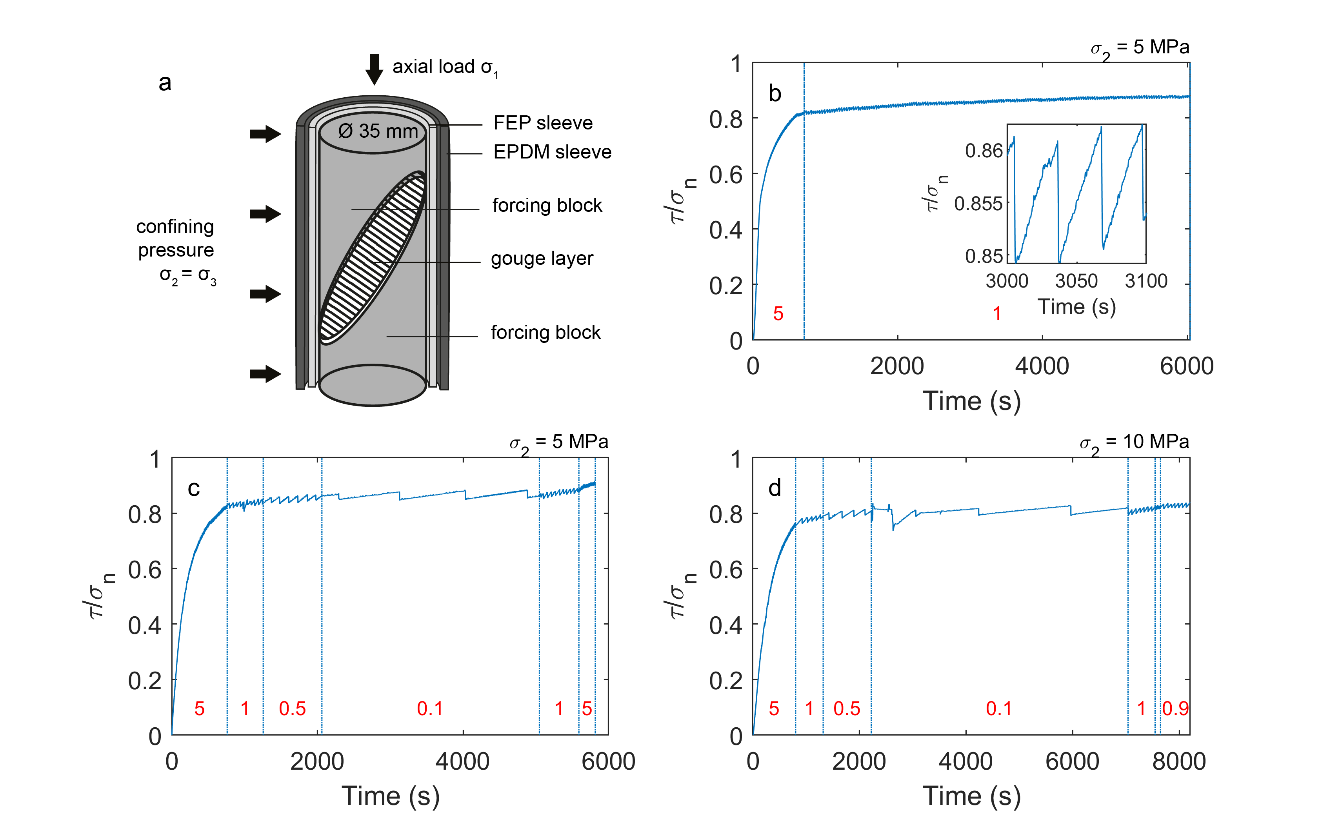


Figure S 1 Friction of gypsum gouge deformed along a saw-cut interface in a triaxial loading apparatus. a) Sample assembly with gouge layer along saw-cut interface. b and c) Friction measured during shearing of the gypsum gouge layer. Two separate experiments are shown, both with a confining pressure of 5 MPa. Red numbers indicate the imposed load point velocity in µm/s, which range from 0.1 to 5 µm/s. The gouge was the same as the gypsum gouge used in the biaxial experiments described in the main document, and had equilibrated to a relative humidity of 75%. d) Friction measured during shearing of gypsum gouge at a confining pressure of 10 MPa.

1. Friction and velocity-dependence of gypsum gouge from rotary shear experiments

Gypsum gouge was also sheared in a rotary shear apparatus (see apparatus description in van den Ende & Niemeijer, 2019). The gouge had been equilibrated to a relative humidity (RH) of 75%. The gouge was gripped by two steel rings with an inner diameter of 80 m and an outer diameter of 100 mm. The applied normal stress was varied between 2 and 10 MPa, and the sliding velocity between 5 and 1000 μms^-1^. Due to the higher stiffness of the rotary shear apparatus with respect to the triaxial apparatus, stable sliding occurred and the velocity steps (for velocities <= 100 μms^-1^) could be modeled with the rate-and-state friction formulation. Additional velocity steps on gypsum gouge at velocities between 300 and 1000 μms^-1^ at 10 and 13.5 MPa normal stress were performed in a biaxial apparatus (Marone, pers. comm.). These required a two-state variable friction law. Nucleation lengths for these data were computed using b_1_ and D_c1_.


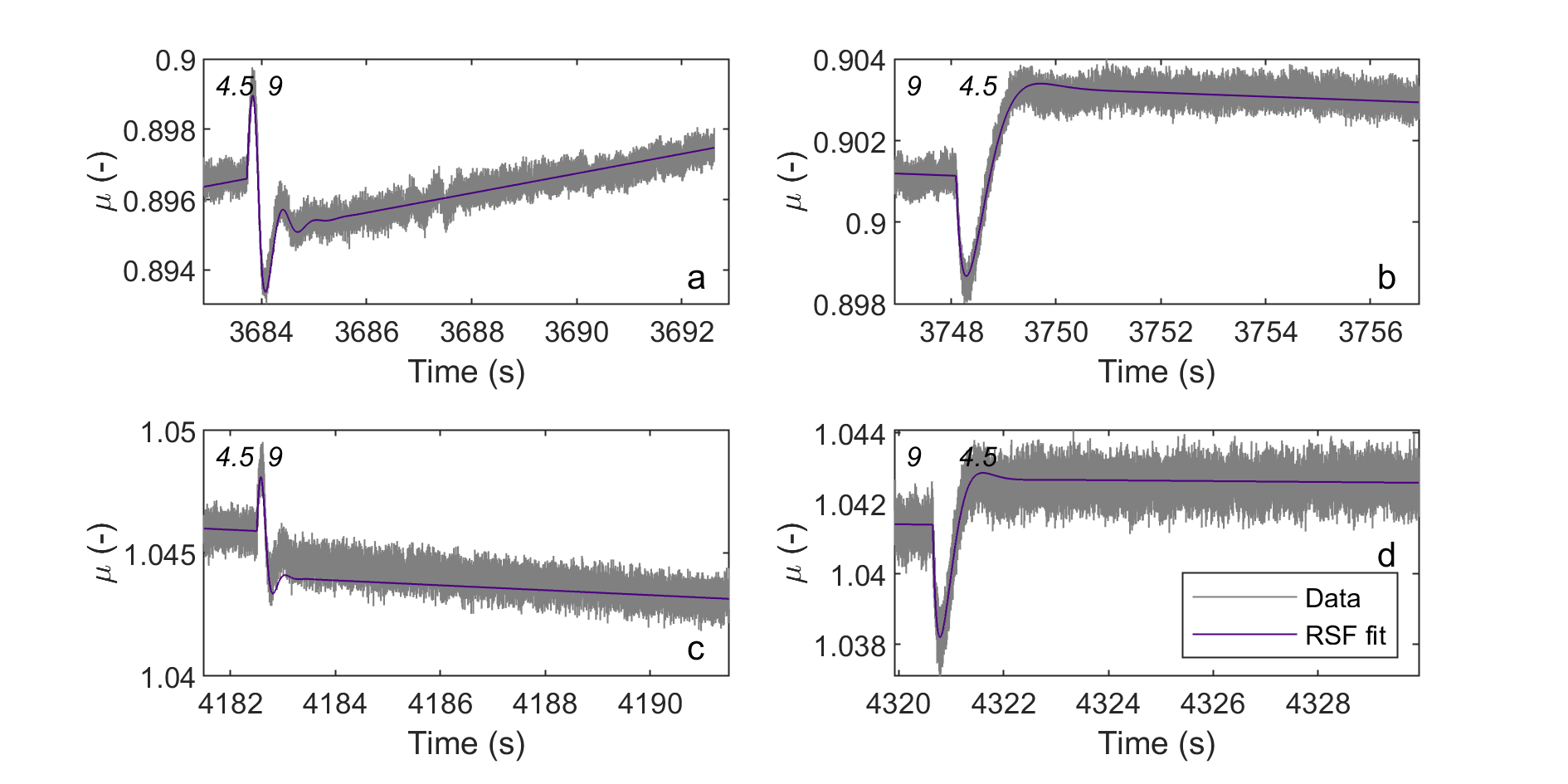


Figure S 2 Velocity steps performed on gypsum gouge in the rotary shear apparatus and rate-and-state (RSF) fits. The RSF fits are shown for the aging law. Italic number indicate step velocity in μms^-1^. a) and b) were performed at σ_n_ = 10 MPa, c) and d) at σ_n_ = 5 MPa.


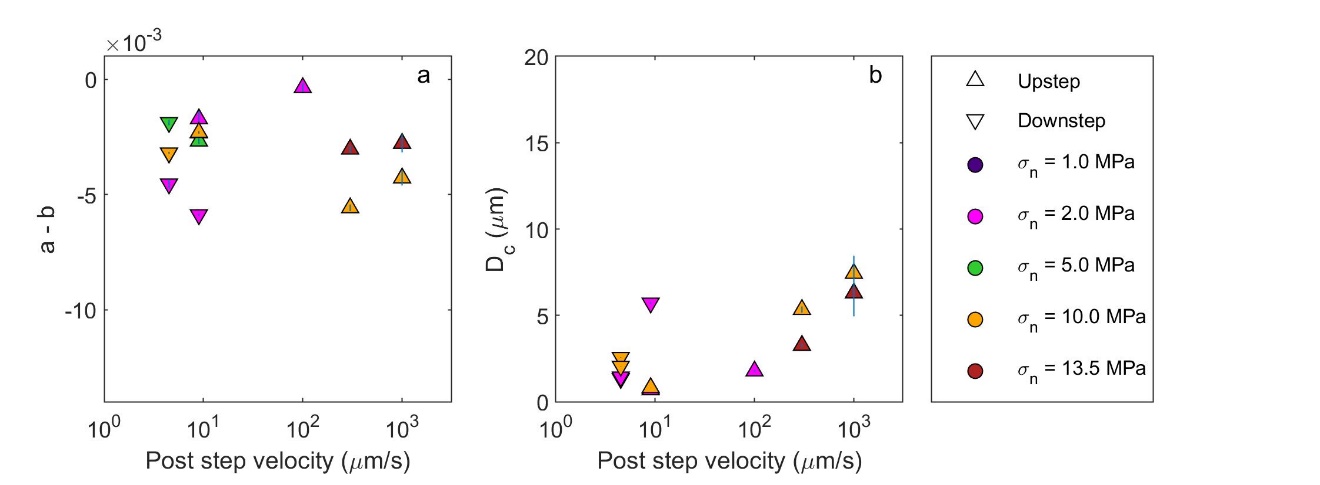


Figure S 3 RSF parameters obtained from fits of velocity step on gypsum gouge (aging law).


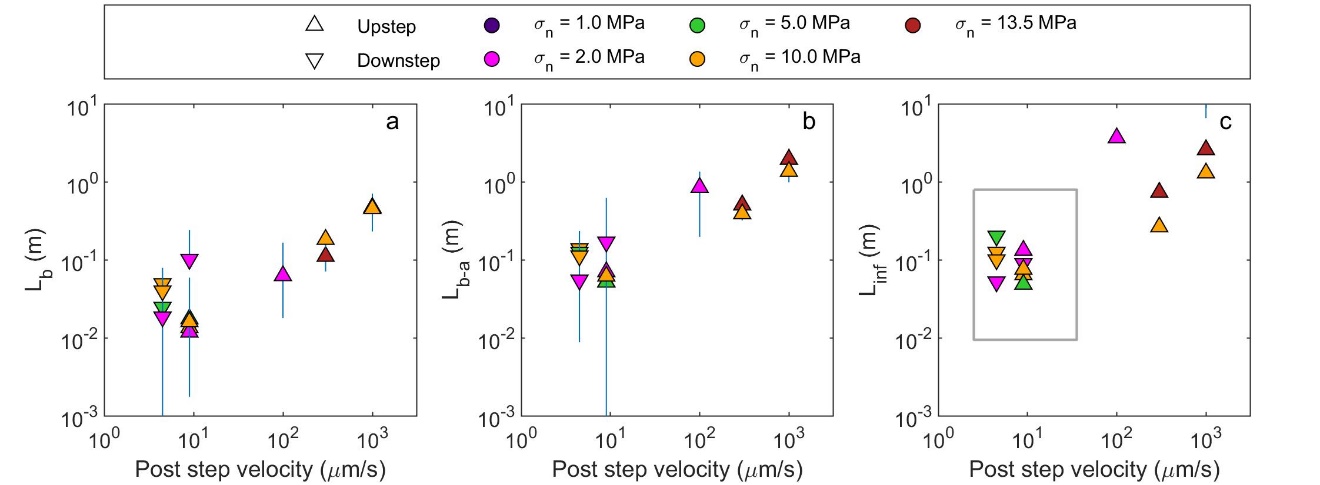


Figure S 4 Nucleation lengths calculated from RSF parameters (see Section 1.1)

1. Calculation of shear and normal strains and stresses from strain gauge data


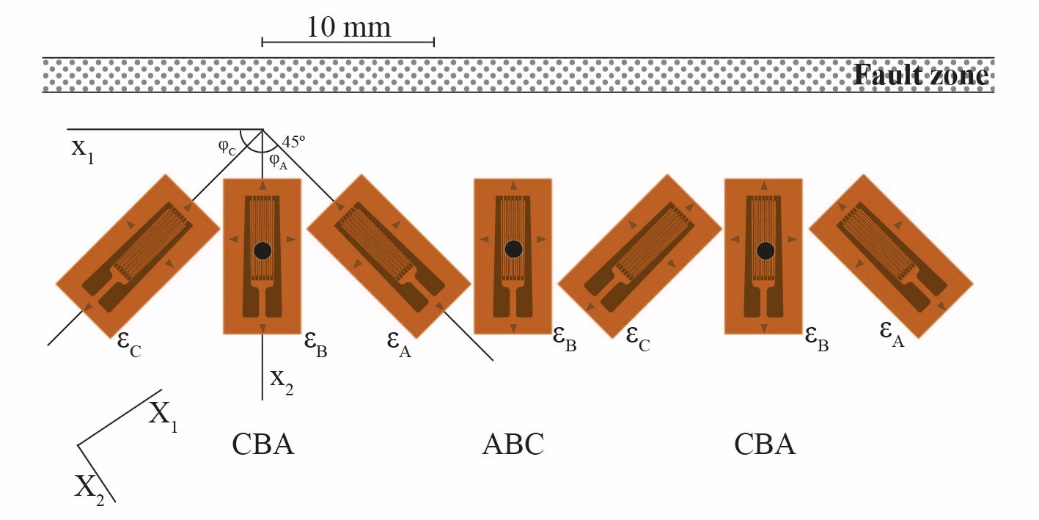


Figure S 5 Planar rectangular strain gauge rosette setup with respect to the fault zone (view from the top of the sample). Local axis x_1_ and x_2_ are defined perpendicular and parallel to the fault. Strain gauges A and C are positioned at a 45 degree and 135 angle to x_1_ respectively, whereas B is perpendicular to x_1_. X_1_ and X_2_ indicate the global axes along which loading (σ_1_ and σ_2_) was applied.

Strain gauges were installed to record local strains along the fault margin. The strain gauges were placed on the bottom of one of the PMMA fault blocks 10 mm from the fault margin (Figure 1). The gauges were placed in a planar rectangular setup with three different orientations with respect to the local principal axis x_1_ (A = 135º, B = 90º, C = 45º). The strains in the gauges A, B, and C with respect to the local principal axes *x_1_* and x_2_ can be expressed in local principal strains ε_1_ (fault-normal) and ε_2_ (fault-parallel) as follows:

| $\varepsilon_{A,B,C}=\varepsilon_{1}\cos^{2} \varphi+ \varepsilon_{2}\sin^{2} \varphi+\gamma_{12}sin\varphi cos\varphi,$ | S1 |
| --- | --- |

where φ is the angle with respect to *x_1_*. Rewriting Equation gives

| $\varepsilon_{A,B,C}=\frac{\varepsilon_{1}+\varepsilon_{2}}{2}+\frac{\varepsilon_{1}-\varepsilon_{2}}{2}\cos2\varphi+\frac{\gamma_{12}}{2}sin2\varphi,$ | S2 |
| --- | --- |

which gives the strain in gauge A (*φ_A_* = 135°)

| $\varepsilon_{A}=\frac{\varepsilon_{1}+\varepsilon_{2}}{2}-\frac{\gamma_{12}}{2},$ | S 3 |
| --- | --- |

the strain in gauge B (*φ_B_* = 90°)

| $\varepsilon_{B}=\varepsilon_{2},$ | S 4 |
| --- | --- |

and the strain in gauge C (*φ_C_* = 45°)

| $\varepsilon_{C}=\frac{\varepsilon_{`1}+\varepsilon_{2}}{2}+\frac{\gamma_{12}}{2}.$ | S 5 |
| --- | --- |

Substituting S3, S4, and S5 gives the principal strain in the *x_1_* direction

| $\varepsilon_{1}=\varepsilon_{`C}-\varepsilon_{B}+\varepsilon_{A},$ | S 6 |
| --- | --- |

and shear strain

| $\gamma_{12}=\varepsilon_{C}-\varepsilon_{A}.$ | S 7 |
| --- | --- |

for setup CBA, or

| $\gamma_{12}=\varepsilon_{A}-\varepsilon_{C}.$ | S 8 |
| --- | --- |

for setup ABC.

The stress-strain relationship for 2D plane-stress (the bottom of the block is a free surface) is given by

| $\left\lceil\begin{matrix} \sigma_{1} \\ \sigma_{2} \\ \tau_{12} \end{matrix} \right\rceil=\left[ \begin{matrix} E/(1-\nu^{2}) & E\nu/(1-\nu^{2}) & 0 \\ E\nu/(1-\nu^{2}) & E/(1-\nu^{2}) & 0 \\ 0 & 0 & G \end{matrix} \right]\left\lceil\begin{matrix} \varepsilon_{1} \\ \varepsilon_{2} \\ \gamma_{12} \end{matrix} \right\rceil$ | S 9 |
| --- | --- |

where *E* is Young’s modulus, *ν* is Poisson’s ratio, and *G* is the shear modulus.

The local shear stress along the fault *τ* in the area covered by the strain gauge rosette is

| $\tau=G\gamma_{12}.$ | S 10 |
| --- | --- |

and the local normal stress *σ_n_* which is parallel to x_2_ is given as

| $\sigma_{n}=\frac{E}{1-\nu^{2}}\left( \varepsilon_{2}+\nu\varepsilon_{1} \right).$ | S 11 |
| --- | --- |

The stresses computed from each rosette are given at the location of the center strain gauge (gauge B).

1. Finite Element model used to compute fault stresses

A Finite Element model was constructed to help interpret the state of stress on the fault. We focus on the stress developing during early deformation and the onset of slip. The PMMA forcing blocks, fault zone, and steel spacers were modeled using DIANA Finite Element Analysis v10.1 (DIANA 10.1 User Manual, 2016). The geometry of the PMMA blocks was identical to that in the experimental setup (Figure 1b). Both 3D model and a 2D plane stress model were constructed (Figure S6). Steel spacers with a thickness of 25 mm (3D model) or 100 mm (2D model) were included in the model on each side. Linear elasticity was assigned to the PMMA blocks and the steel spacers, with elastic moduli for the PMMA equal to the moduli of PMMA used in the experiments (*E*: 3.3 GPa, *ν*: 0.37, *ρ*: 1190 kgm-3), and with representative elastic moduli for the steel spacers (*E*: 200 GPa, *ν*: 0.3, ρ: 7600 kgm-3). The PMMA blocks and steel spacers were modeled using linear solid elements with an unstructured mesh which was refined close to the fault zone (Figure S6a).

Five frictional interfaces were included in the 3D model, representing the fault zone and the four interfaces between the steel spacers and PMMA forcing blocks. The fault and steel-PMMA interfaces were modeled using planar rectangular interface elements. The strength of these interfaces was governed by Mohr-Coulomb friction

| $\tau_{f}=C+\sigma_{n}\mu,$ | S 12 |
| --- | --- |

where *σ_n_* is the fault normal stress, and *C* and *µ* are the appropriate cohesion and friction coefficient. For the fault a friction coefficient of 0.78 was assumed, similar to that measured in small scale experiments. The steel-PMMA interface was also modeled as a frictional interface. Here a steel-PMMA friction coefficient *μ_SIF_* of 0.3 was prescribed, after measurements obtained from conventional triaxial tests where PMMA was sheared against steel along a sawcut interface. For the 3D model roller boundaries were included to support two of the steel spacers. These rollers prevent motion in the x1-direction (short side) and x2-direction (long side), but allow motion in the respectively x2- and x1-direction. A constant stress load or constant displacement load was applied to the other two steel spacers.


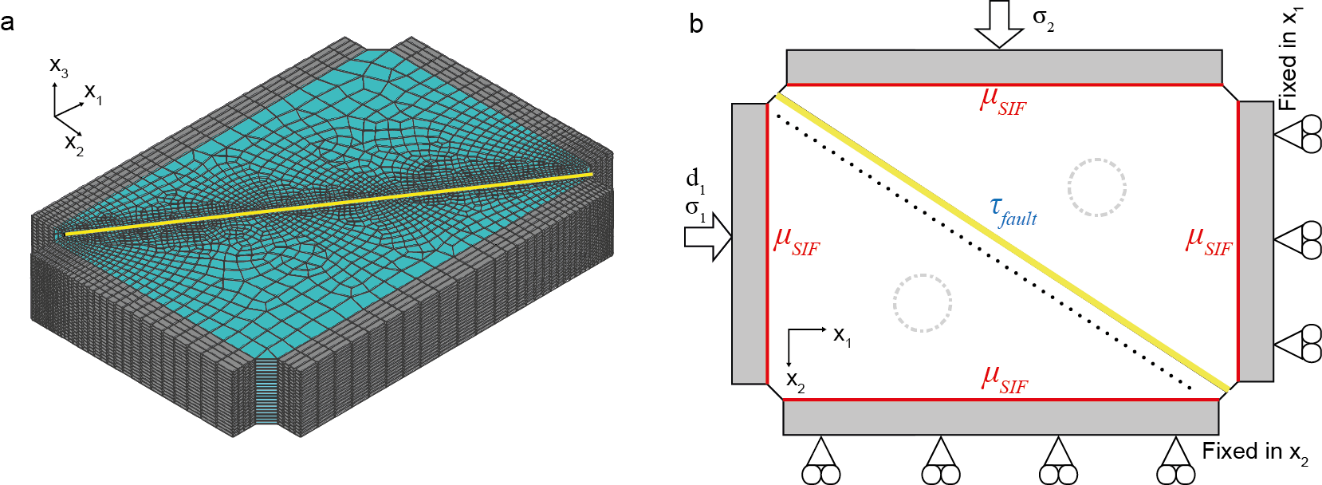


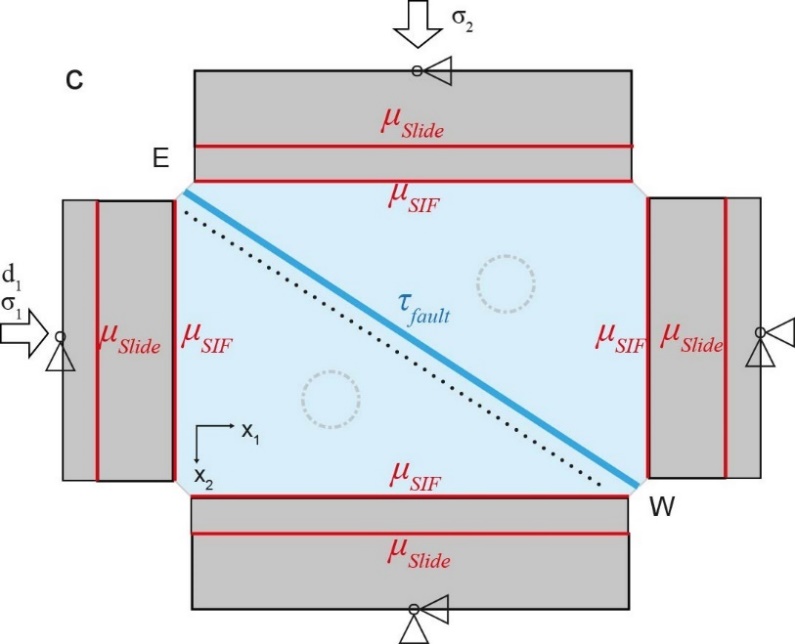


Figure S 6 Finite Element model setup. a and b show the 3D model setup (model setup A), with a) example of mesh of the PMMA forcing blocks and fault. b) Top view showing the experimental geometry and the imposed boundary conditions. Stresses σ_1_ and σ_2_ indicate the load-controlled boundary conditions, and d_1_ displacement-controlled boundary conditions. Triangles indicate roller boundaries preventing motion in either x_1_ or x_2_, whilst allowing motion in respectively x_2_ and x­_3_ or x_1_ and x_3_. The dots along the fault indicate the position of strain measurements on the bottom of the PMMA block, agreeing with the strain gauge positions in the experiment. c) Model setup B: plane stress model setup including slide bearing plates and rotation degrees of freedom of the steel spacers.

For the 2D model the same five frictional interfaces (fault, steel-PMMA) were present, but in addition also four slide-bearing plates were included with low friction of µ_slide_ = 0.15 (Figure S6c), as in the experiment (Figure 1a). The loads and supports were applied to the center of the steel spacers. The supports prevent translation (except in the direction of the loads), but allow rotation of the steel spacers.

The elastic behavior of the interfaces was controlled by a normal and shear stiffness *k_n_* and *k_t_*. High stiffnesses of 100 GPA/m were assigned to model a stiff fault (e.g. for thin fault zones such as in the bare PMMA fault experiment). For the compliant fault a value of *k_n_* was chosen such that the elastic fault-normal deformation matched the fault normal compaction observed from DIC measurements at the start of shearing (*k_n_* = 10 GPa/m). The shear stiffness of the gypsum was assumed 10 times larger.

The model procedure started with initialization of gravitational body stresses. Second, the sample was loaded biaxially to 5 MPa. Third, a fixed displacement rate was prescribed parallel to x1 and the stress accordingly increased until first activation of slip in the gypsum fault.

The figures below show respectively the effect of allowing/preventing rotation of the steel pistons (Figure S7), the effect of friction between the PMMA and the steel spacers (Figure S8), and the effect of the compliance of the fault zone (Figure S9).


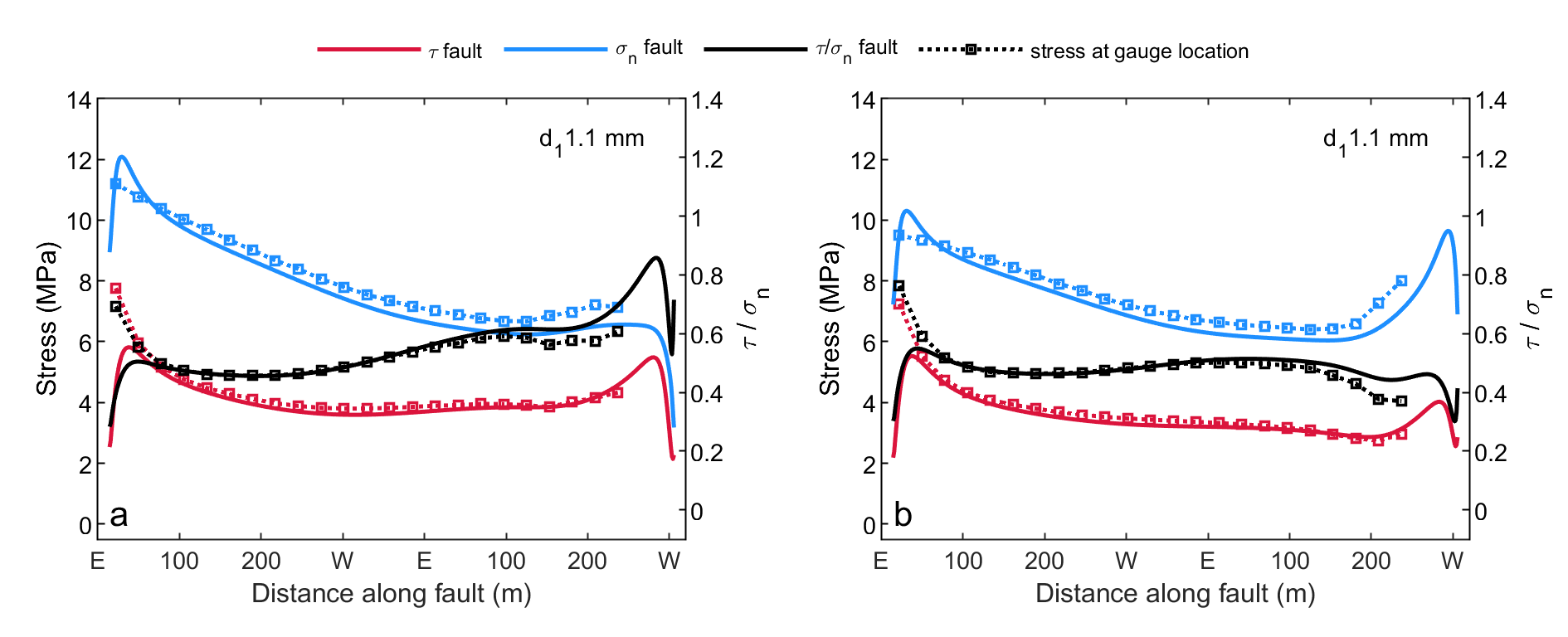


Figure S 7 Effect of allowing rotation of steel spacers on fault stresses. a) Fault stresses for a scenario where rotation of steel spacers around vertical axis is allowed as in Figure S6c. b) Fault stresses for a scenario where rotation of steel spacers around vertical axis prohibited.


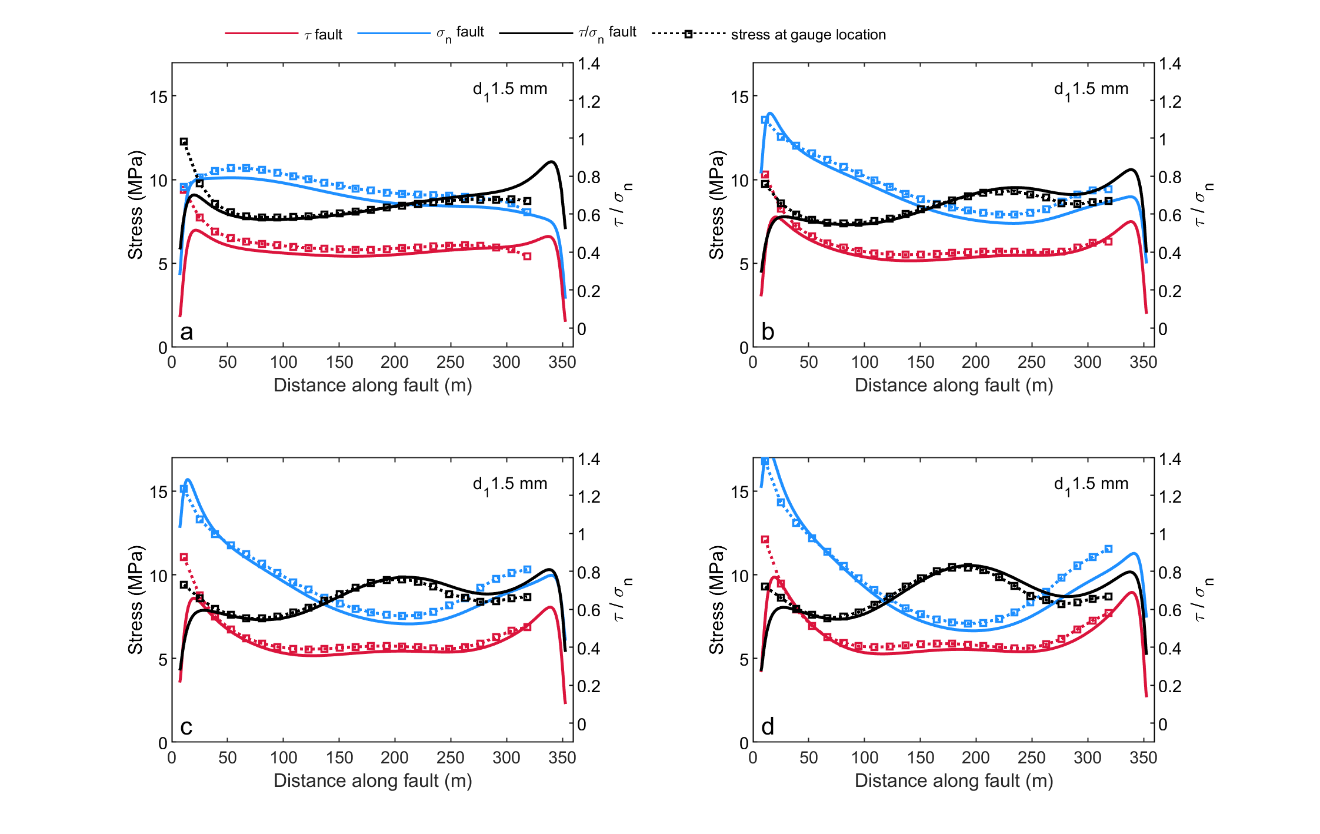


Figure S 8 Effect of PMMA-steel interface friction on fault stresses. a) μ_SIF_ = 0.1, a) μ_SIF_ = 0.4, a) μ_SIF_ = 0.6, d) μ_SIF_ = 0.9. Load point displacement d_1_ as indicated.


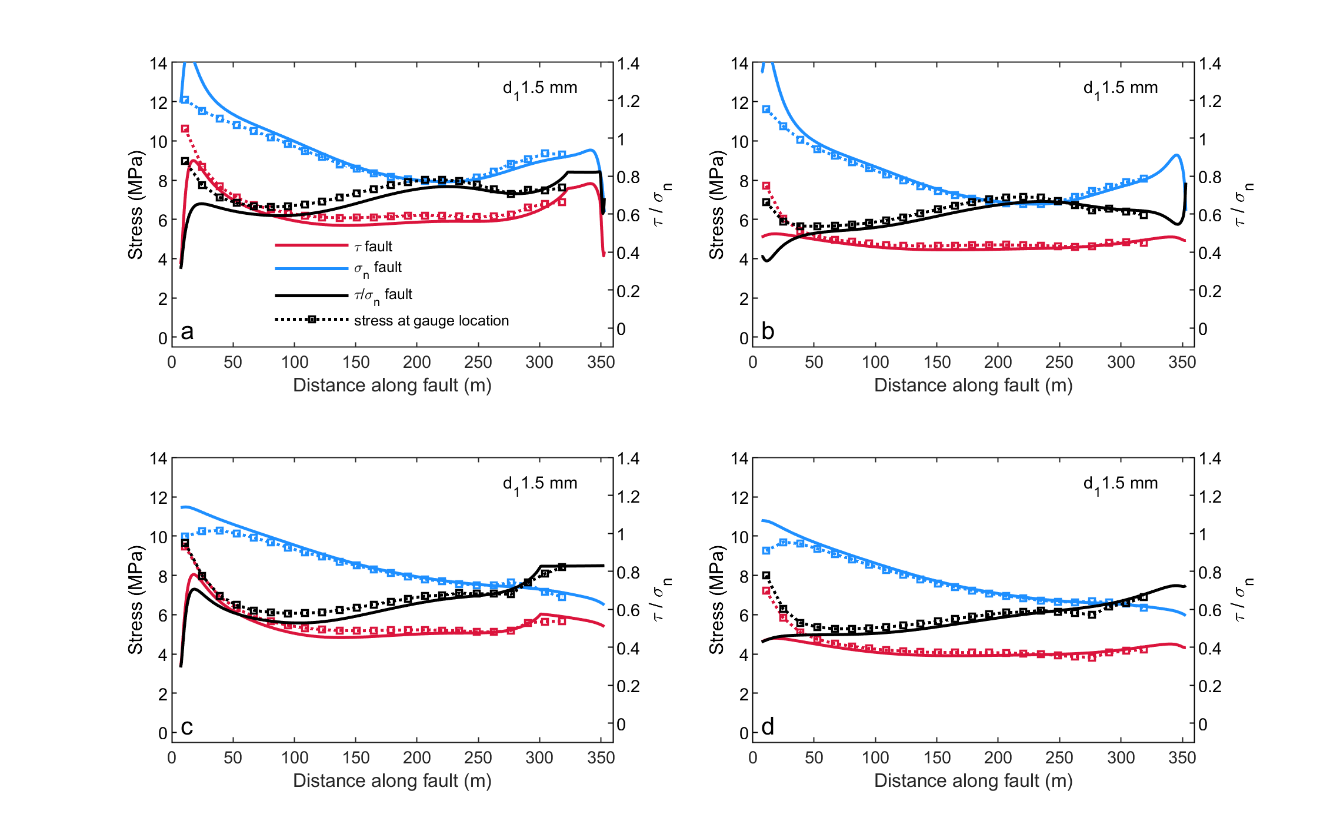


Figure S 9 Effect of elastic deformation of the fault zone on fault stresses. Fault stresses for different values of normal stiffness *k_n_* and shear stiffness *k_t_* are shown at a load point displacement d_1_ of 1 mm. a) stiff fault with a normal and shear stiffness of 1000 GPa/m, b) fault zone that is compliant (10 GPa/m) in the shear direction and stiff in the normal direction (1000 GPa/m), c) fault zone that is stiff (1000 GPa/m) in the shear direction and compliant (10 GPa/m) in the normal direction, d) compliant fault (10 GPa/m).

1. The effect of gouge loss on the normal stress recorded by the strain gauges

Visual inspection of the gouge layer and PMMA forcing blocks after the experiment showed that parts of the gouge layer were cohesive, and compacted gouge stuck to the PMMA over most of the fault surface. However, in a zone of several mm along the fault margins the gouge was still powdery, and no gouge remained stuck to the fault surface, indicating that the gouge may locally have been extruded, and/or too loose to support the load and compact. This can affect the stresses measured by the strain gauges, which are placed at 10 mm from the fault at the bottom surface of one of the PMMA blocks. To evaluate the (potential) effects of gouge loss on the strain measurements the Finite Element model (model setup A) was adapted to include a gouge layer of a certain height. To simulate the effect of gouge loss, an endmember model was built where 5 mm at the top and bottom of the fault zone did not contain any gouge (Figure 10a). This endmember case was compared to the base case where the gouge layer was present over the entire surface.


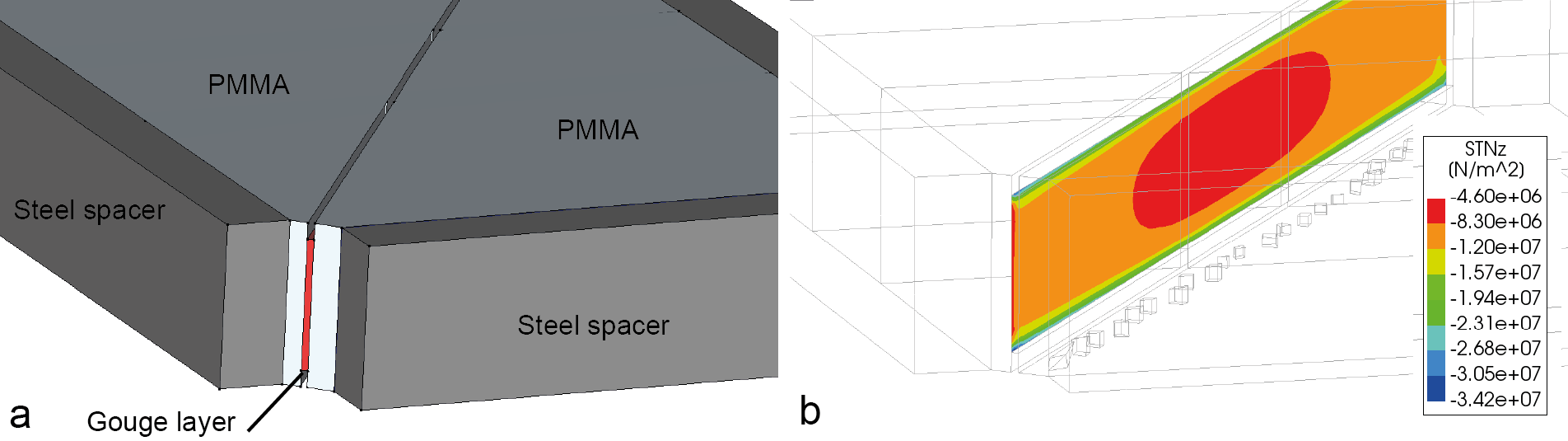


Figure S 10 Effect of gouge loss on fault stresses. a) Model geometry for evaluating the effect of gouge loss on recorded stresses at the strain gauge locations. b) Fault normal stress after 1 mm of load point displacement.

The absence of gouge along the fault margins had a strong effect on the normal stress distribution on the fault and the normal stresses recorded at the strain gauge locations. At the fault center the normal stress was similar to the base case scenario without gouge loss (~ =8 MPa) but at the top and bottom margins of the gouge layer the normal stress was concentrated (Figure S11b). In the PMMA blocks along the top and bottom margins of the fault the normal stress decreased strongly, as near the top and bottom no gouge layer was present to support the normal stress. The modeled stress at the strain gauge locations was about 50% of the normal stress at the fault center (Figure S8b). The shear stress modeled at the strain gauge location was less affected by the absence of gouge along the fault margins. It was 10 – 20% lower than the shear stress at the center of the fault. Consequently, the modeled stress ratio at the strain gauge locations was significantly higher than the stress ratio acting on the fault and exceeded 1.


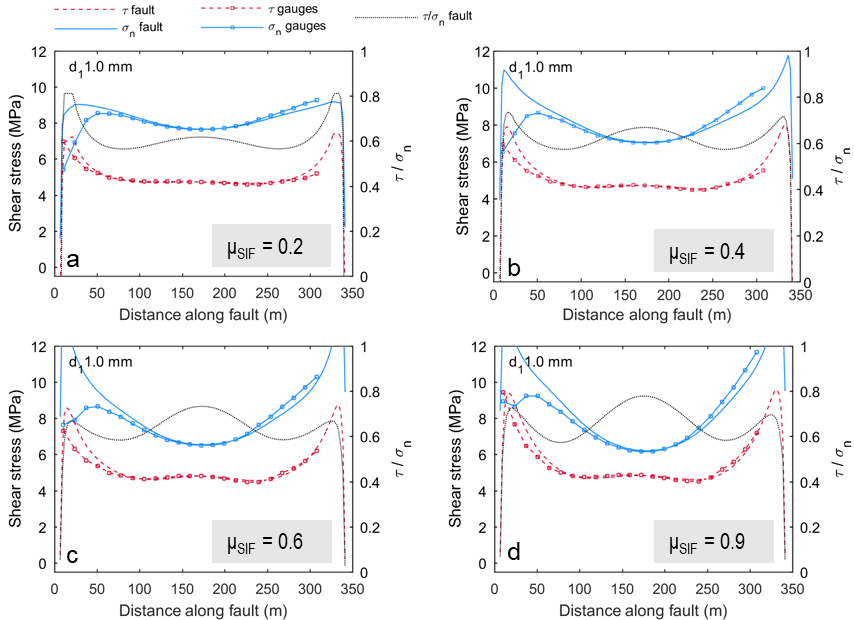


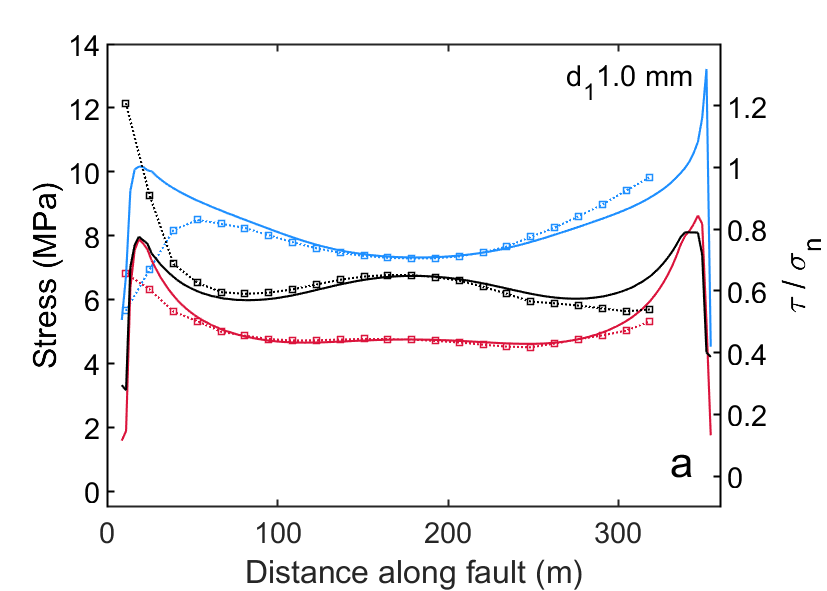

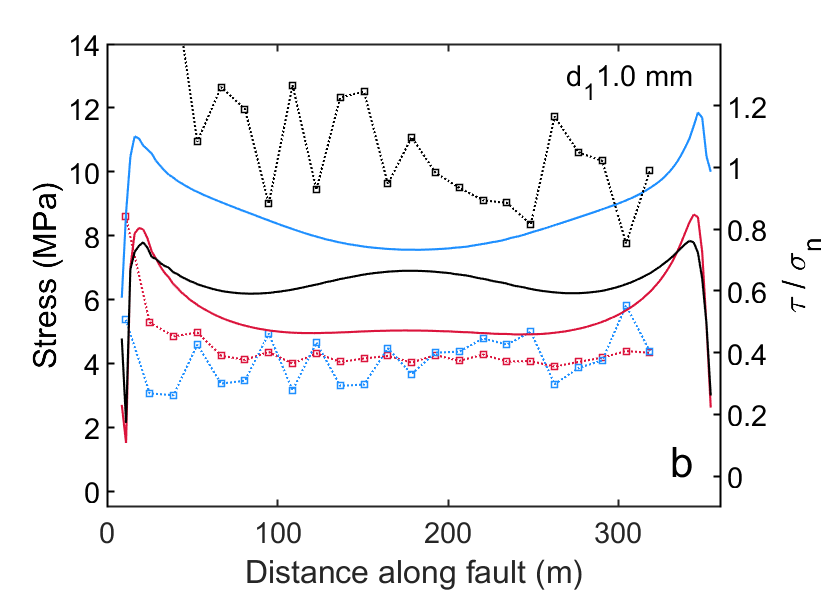


Figure S 11 Effect of gouge loss on the stresses recorded at the strain gauge locations. Confining pressure σ2 = 5MPa and the load point displacement is 1 mm. For definition of lines and symbols see Figure S 8. a) Endmember case without gouge loss. b) Endmember case where 5 mm along the fault margins the gouge layer is absent.
